# Defect‐Driven Reconstruction of Bismuth Nanoflowers via Precursor Engineering for Highly Efficient CO_2_‐to‐Formate Electrochemical Reduction

**DOI:** 10.1002/smsc.202500296

**Published:** 2025-08-20

**Authors:** Jiaying Yan, Masao Kamiko, Teruyasu Mizoguchi, Shunsuke Yagi

**Affiliations:** ^1^ Institute of Industrial Science The University of Tokyo 4‐6‐1 Komaba Meguro‐ku Tokyo 153‐8505 Japan

**Keywords:** CO_2_ electrochemical reduction, defect engineering, microstructures, small‐angle grain boundaries, structural evolution

## Abstract

Electrochemical reduction of CO_2_ represents a promising strategy for converting CO_2_ into value‐added chemical products. Currently, significant efforts have been devoted to developing more efficient Bi‐based catalysts to reduce CO_2_ to formate. However, the role of precursor defects in directing the electrochemical reconstruction of Bi‐based catalysts is yet to be thoroughly investigated. Herein, a strong alkali‐induced strategy, coupled with electrochemical reduction for controllable morphology and defect engineering to achieve Bi_2_O_3_ reconstruction with oxygen vacancies (O_v_‐Bi_2_O_3_) into defect‐rich Bi nanoflowers (Bi (O_v_‐Bi_2_O_3_)) featuring small‐angle grain boundaries, is reported. Over a wide potential range (from −0.87 to −1.17 V vs. reversible hydrogen electrode [RHE]) in an H‐type cell, Bi (O_v_‐Bi_2_O_3_) exhibits a high Faradaic efficiency of formate (>95%). Moreover, it displays a high current density (−340 mA cm^−2^) at −1.08 V versus RHE and a formate production rate of 6.09 mmol h^−1^ cm^−2^ in a flow cell, thus highlighting its potential for industrial applications. In situ attenuated total reflectance surface‐enhanced infrared absorption spectroscopy offers detailed insights into the reaction mechanism, verifying that formate formation predominantly occurs through the *OCHO intermediate. This study reveals the role of precursor‐derived defects in catalytic properties, clarifying mechanisms and guiding performance optimization.

## Graphical abstract

A defect‐engineered Bi nanoflower catalyst is developed for CO_2_ reduction to selectively produce formate. The nanoflower catalyst exhibits >95% Faradaic efficiency over a wide potential range and achieves −340 mA cm^−2^ at –1.08 V versus reversible hydrogen electrode in a flow cell.

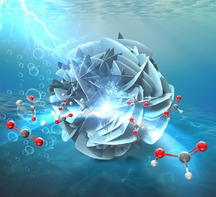

## 1 Introduction

The long‐term reliance on fossil fuels has led to a dramatic rise in atmospheric CO_2_ levels, triggering global climate change and exacerbating a range of environmental challenges.^[^ ^1^, ^2^ ^]^ Thus, clean energy alternatives for replacing traditional fossil fuels that are coupled with efficient CO_2_ utilization strategies for sustainable development are urgently needed.^[^ ^3^, ^4^ ^]^ Hydrogen has recently been recognized as a cornerstone of future green energy systems; however, conventional physical hydrogen storage methods are often costly and involve significant safety risks during transportation.^[^ ^5^ ^]^ Formic acid/formate has emerged as a promising alternative because it enables efficient hydrogen release at both room temperature and ambient pressure and provides a large volumetric hydrogen storage capacity.^[^ ^6^, ^7^, ^8^ ^]^ Moreover, formic acid/formate can be directly produced via the electrochemical reduction of CO_2_ (ERCO_2_) using renewable electricity as the sole energy source.^[^ ^9^, ^10^, ^11^ ^]^ Hence, ERCO_2_ to formic acid/formate is recognized as a potential solution to environmental challenges and a pathway to advancing energy storage technologies.

Numerous catalysts made of post‐transition metals, particularly Bi, Sn, and Pb, have been investigated owing to their high catalytic ability to convert CO_2_ into formate.^[^ ^12^, ^13^ ^]^ Among these, Bi‐based materials have garnered significant attention due to their nontoxicity and cost‐effectiveness.^[^ ^14^, ^15^ ^]^ Mechanistically, the initial electron transfer to *CO_2_ on Bi‐based catalysts to form the *CO_2_^**−**^ intermediate is widely regarded as the rate‐limiting step in ERCO_2_, due to the inherent chemical inertness of CO_2_ molecules.^[^ ^16^ ^]^ Notably, the subsequent protonation pathway diverges based on the surface binding mode of *CO_2_^**−**^, generating two intermediates: *OCHO exclusively leads to formate production, while *COOH can further yield either formate or carbon monoxide.^[^ ^17^ ^]^ Various strategies, including atom doping and alloying, have recently been employed to modulate Bi‐based catalysts toward the preferential formation of the *OCHO intermediate, thereby enhancing formate production efficiency.^[^ ^18^, ^19^, ^20^, ^21^ ^]^

The substantial cathodic polarization required for CO_2_ activation inevitably induces structural transformations in Bi‐based catalysts; therefore, precursor engineering has attracted attention owing to its significant influence on reconstruction pathways and catalytic performance.^[^ ^22^, ^23^ ^]^ For example, Wang et al.^[^ ^24^ ^]^ introduced chlorine in the one‐step hydrothermal synthesis of Bi_19_Cl_3_S_27_ nanowires, which were subsequently electrochemically reduced to Bi nanosheets with atomic vacancies. The resulting nanosheets exhibited high activity and selectivity for CO_2_‐to‐formate conversion. Ma et al.^[^ ^25^ ^]^ reported a Bi_19_Br_3_S_27_‐derived electrocatalyst that undergoes in situ reconstruction into S, Br‐comodified Bi, generating positively charged Bi sites and stabilizing key intermediates, thereby achieving high activity toward formate production. Collectively, a comprehensive understanding of the relationship between electrochemical reconstruction mechanisms and catalytic efficiency is essential for the development of highly active electrocatalysts. However, the role of precursor defects in governing catalyst reconstruction during the electrochemical reduction process remains insufficiently explored. Unlike doping or alloying, which introduces heteroatoms and directly modifies the composition and active sites, defect engineering focuses more on tuning the local coordination environment within the precursor itself, thus influencing the structural reconstruction and catalytic performance of the resulting material.^[^ ^26^, ^27^ ^]^ The intrinsic defects can serve as driving forces or nucleation centers for structural reconstruction, leading to more favorable phase evolution, surface reconstruction, or active‐site exposure during electrochemical reduction.

In this study, we demonstrate the structural transformation from Bi_5_O_7_NO_3_ to oxygen vacancy‐rich Bi_2_O_3_ (denoted as O_v_‐Bi_2_O_3_) with unique morphological features, followed by electrochemical reconstruction to form Bi nanoflowers (denoted as Bi (O_v_‐Bi_2_O_3_)) containing structural defects and small‐angle grain boundaries. Specifically, Bi_5_O_7_NO_3_ undergoes strong alkaline treatment, inducing structural evolution and defect formation to generate O_v_‐Bi_2_O_3_. The formed oxygen vacancies facilitate extensive surface reconstruction during the electrochemical reduction process, maximizing the exposed active sites and enhancing key intermediate adsorption on the Bi (O_v_‐Bi_2_O_3_) catalyst. The ERCO_2_ performance was evaluated in both the H‐type cell and flow cell. In the H‐type cell, Bi (O_v_‐Bi_2_O_3_) exhibited a high Faradaic efficiency of formate formation (FE_formate_) exceeding 95% over a wide potential range from −0.87 to −1.17 V versus reversible hydrogen electrode (RHE). Meanwhile, in the flow cell, Bi (O_v_‐Bi_2_O_3_) achieved a current density of 340 mA cm^−2^ at −1.08 V versus RHE with a corresponding formate formation rate of 6.09 mmol h^−1^ cm^−2^, demonstrating considerable potential for industrial‐scale applications. Moreover, the ex situ Raman spectroscopy revealed that the presence of oxygen vacancies significantly accelerates the structural evolution of Bi_2_O_3_. Furthermore, in situ attenuated total reflectance surface‐enhanced infrared absorption spectroscopy (ATR‐SEIRAS) provided insights into the ERCO_2_ mechanism on the Bi (O_v_‐Bi_2_O_3_) catalyst, confirming that formate formation primarily occurred via the *OCHO intermediate.

## 2 Results and Discussion

### 2.1 Structural Characterization of Catalysts

**Figure 1:**
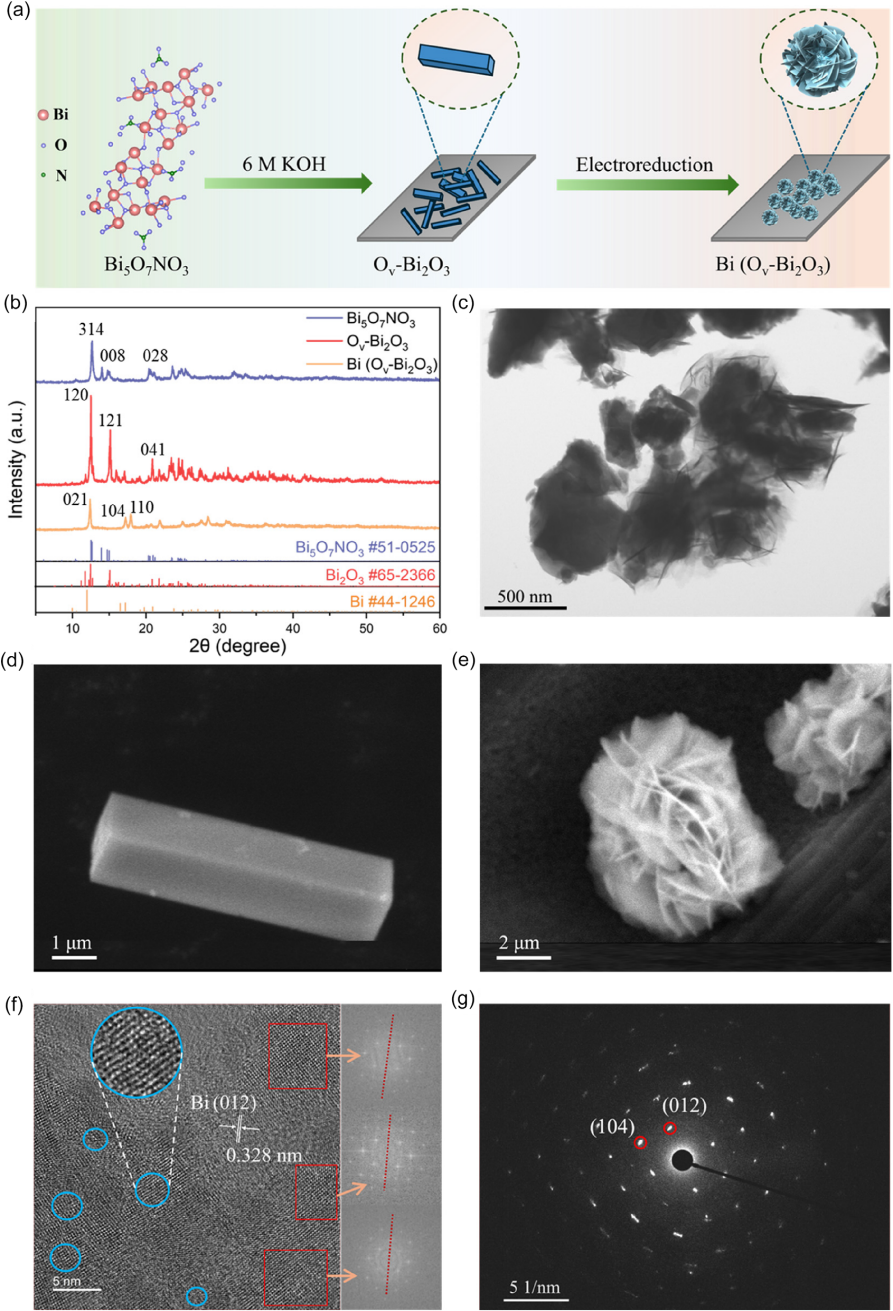
Synthesis procedure and structure characterizations. a) Schematic representation of the fabrication procedure for Bi (O_v_‐Bi_2_O_3_). b) XRD patterns of Bi_5_O_7_NO_3_, O_v_‐Bi_2_O_3_, and Bi (O_v_‐Bi_2_O_3_) samples measured with Mo *K*α radiation. c) HAADF–STEM image of Bi_5_O_7_NO_3_, d) SEM image of O_v_‐Bi_2_O_3_. e) SEM image of Bi (O_v_‐Bi_2_O_3_). f) HRTEM image of Bi (O_v_‐Bi_2_O_3_), and corresponding FFT patterns of the three adjacent red‐marked regions shown on the right. g) Corresponding SAED pattern of Bi (O_v_‐Bi_2_O_3_).

As illustrated in **Figure** **1**a and S1, Supporting Information, Bi_5_O_7_NO_3_ was initially synthesized as the precursor, and its phase structure was characterized by X‐ray diffraction (XRD), confirming the formation of a pure Bi_5_O_7_NO_3_ phase (PDF # 51‐0525) without any detectable impurity peaks (Figure 1b). The morphologies and microstructure were then observed using high‐angle‐annular dark‐field–scanning transmission electron microscopy (HAADF‐STEM) with energy‐dispersive spectroscopy (EDS). As shown in Figure 1c, Bi_5_O_7_NO_3_ exhibits a morphology in which sheet‐like precipitates were loosely aggregated. The EDS elemental mapping in Figure S2a, Supporting Information, shows the uniform distribution of Bi and O in Bi_5_O_7_NO_3_, with a weaker N signal corresponding to the presence of NO_3_^**−**^ species. High‐resolution TEM (HRTEM) image in Figure S2b, Supporting Information, displays distinct lattice fringes with a spacing of 0.291 and 0.193 nm, corresponding to the 008 and 028 planes of Bi_5_O_7_NO_3_, respectively. O_v_‐Bi_2_O_3_ sample was synthesized by dispersing the as‐prepared Bi_5_O_7_NO_3_ sample in 20 mL of 6 M KOH aqueous solution with vigorous stirring at room temperature for 5 min. The diffraction peaks of the O_v_‐Bi_2_O_3_ sample at 12.51°, 15.04°, and 20.85° match the 120, 121, and 041 peaks of reference monoclinic Bi_2_O_3_ (#65‐2366), indicating that Bi_5_O_7_NO_3_ undergoes a phase transformation upon reaction in 6 M KOH aqueous solution, ultimately converting entirely into the monoclinic Bi_2_O_3_ phase (Figure 1b). This conversion process in a strong alkaline solution at room temperature involves a sequence of clear chemical and structural transformations; initially, poorly soluble Bi_5_O_7_NO_3_ undergoes hydrolysis in the presence of concentrated hydroxide ions (OH^−^), leading to the dissociation of nitrate ions (NO_3_^−^) to form Bi(OH)_3_ as an intermediate phase. The Bi(OH)_3_ intermediate phase subsequently undergoes spontaneous dehydration to the form Bi_2_O_3_ as shown in Figure 1b. Moreover, as depicted in the scanning electron microscopy (SEM) image (Figure 1d), O_v_‐Bi_2_O_3_ exhibited a rod‐like morphology with sharp edges and facets, indicating a high crystallinity and a controlled and directional growth process. The HRTEM image in Figure S3a, Supporting Information, further corroborates this structural evolution, revealing distinct lattice fringes with a spacing of 0.325 nm, corresponding to the 120 plane of Bi_2_O_3_. In addition, the EDS elemental mapping image of O_v_‐Bi_2_O_3_ revealed a heterogeneous O distribution, unlike the uniform dispersion of Bi (Figure S3b–d, Supporting Information), suggesting the presence of oxygen‐deficient regions potentially associated with oxygen vacancies. Based on these structural characterizations, it is inferred that relatively stable low‐energy planes, such as the (120) plane of Bi_2_O_3_, tend to be preserved and exposed, ultimately leading to the development of stable and anisotropic morphology. In contrast to the irregularly aggregated bulk morphology of commercial Bi_2_O_3_ (I‐Bi_2_O_3_), the alkali‐mediated approach used in this study is a simple and effective route for synthesizing Bi_2_O_3_ with controlled morphology (Figure S4, Supporting Information).

Electrochemical reduction was subsequently conducted at −1.07 V versus RHE for 1 h. The XRD pattern of the sample depicted in Figure 1b indicates the full disappearance of the Bi_2_O_3_ phase and the emergence of the peaks at 12.41°, 17.22°, and 17.95° corresponding to 012, 104, and 110 peaks for metallic Bi (PDF # 44‐1246). The SEM image in Figure 1e exhibits that the resulting Bi (O_v_‐Bi_2_O_3_) has a flower‐like nanostructure with thin, layered nanosheets extending outward. This structure is hierarchically assembled and has a high surface area and porosity, which is favorable to facilitate the adsorption and transport of CO_2_. The transmission electron microscopy (TEM) images further highlighted the layered structure composed of nanosheets with numerous nanopores distributed across the nanosheets (Figure S5, Supporting Information). The HRTEM image in Figure 1f shows well‐defined lattice fringes with a spacing of 0.328 nm, corresponding to the (012) plane of metallic Bi. Additionally, the local fast Fourier transform (FFT) patterns shown on the right‐hand side in Figure 1f revealed a misorientation angle of ≈3° among the adjacent three red‐marked regions, suggesting the presence of small‐angle grain boundaries. Furthermore, the distorted lattice fringes in the blue‐marked region indicate the residual strain possibly induced during the formation process of Bi (O_v_‐Bi_2_O_3_). The presence of the localized strain and small‐angle grain boundaries can also be confirmed in the selected‐area electron diffraction (SAED) pattern with the elongated and slightly diffused diffraction spots of the rhombohedral structure of Bi (O_v_‐Bi_2_O_3_) as shown in Figure 1g. Meanwhile, Bi (I‐Bi_2_O_3_) has a large, agglomerated bulk structure, which may result in few active sites (Figure S6, Supporting Information).

**Figure 2:**
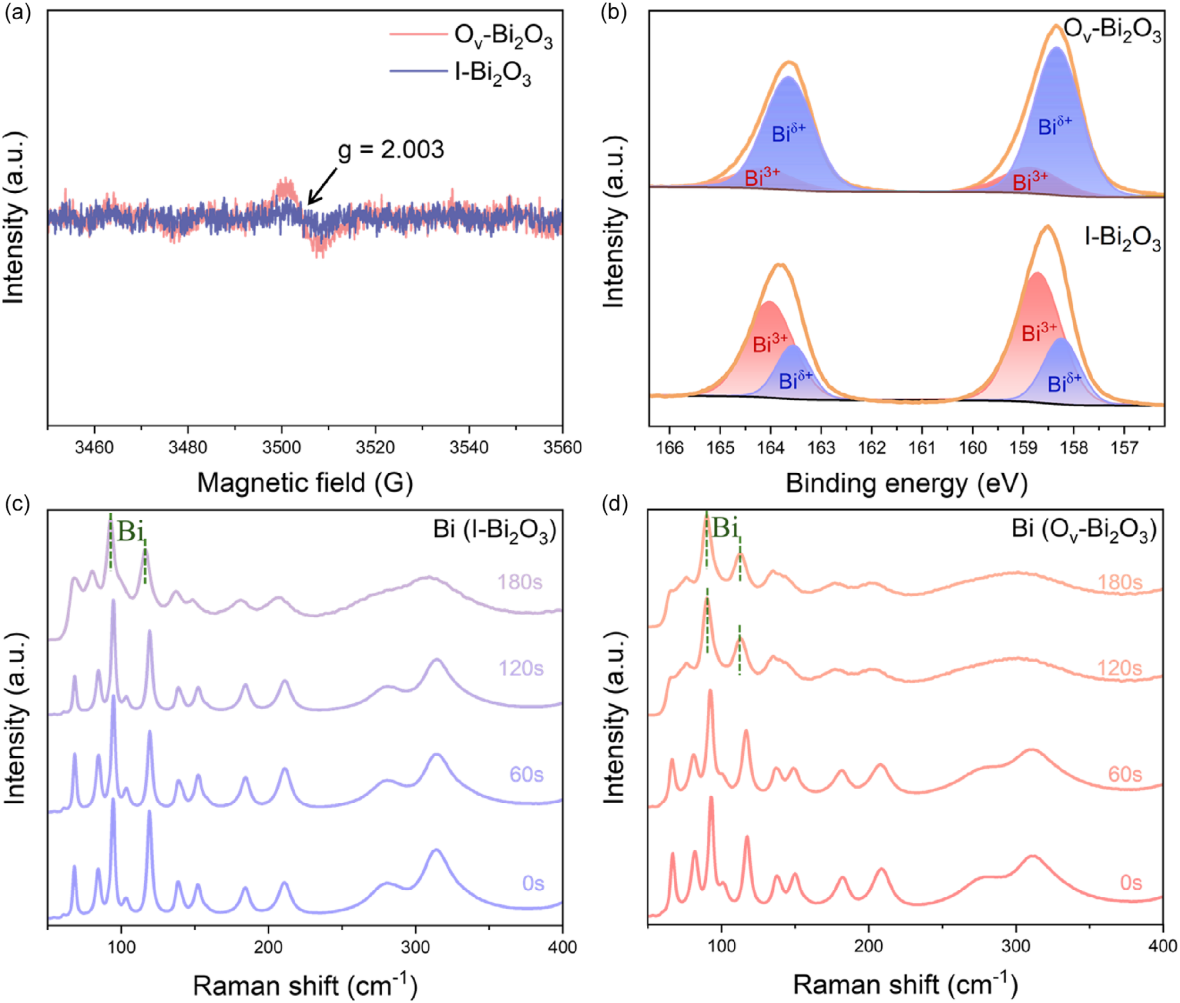
a) EPR spectra and b) high‐resolution XPS spectra at Bi 4f regions of O_v_‐Bi_2_O_3_ and I‐Bi_2_O_3_. Ex situ Raman spectra for c) I‐Bi_2_O_3_ and d) O_v_‐Bi_2_O_3_ collected after varying durations of ERCO_2_ at −1.07 V versus RHE.

Electron paramagnetic resonance (EPR) spectroscopy was performed for I‐Bi_2_O_3_ and O_v_‐Bi_2_O_3_ to investigate structural defects, which are a possible reason for the residual strain. As shown in **Figure** **2**a, I‐Bi_2_O_3_ does not show a distinct signal, while O_v_‐Bi_2_O_3_ displays a signal at ≈3505 G with a *g* factor of 2.003, suggesting the presence of the oxygen vacancies with unpaired electrons.^[^ ^28^ ^]^ As shown in Figure S7, Supporting Information, the spin concentration of 5.5 × 10^11^ spins mg^−1^ was determined for O_v_‐Bi_2_O_3_ by the integration of the fitted EPR signal, corresponding to the concentration of oxygen vacancies in the sample. In contrast, the EPR spectrum of O_v_‐Bi_2_O_3_ showed no detectable signal after spectral fitting, indicating the absence of a measurable concentration of oxygen vacancies. It should be noted that in our preliminary experiment, commercial Bi_2_O_3_ did not show any detectable EPR signal either after the immersion in 6 M KOH aqueous solution for 5 min with vigorous stirring. Therefore, the starting material Bi_5_O_7_NO_3_ is necessary to form O_v_‐Bi_2_O_3_ with oxygen vacancies.

X‐ray photoelectron spectroscopy (XPS) was employed to investigate the impact of oxygen vacancies on the chemical state and coordination environment of Bi. As shown in Figure 2b, the Bi 4f spectra of I‐Bi_2_O_3_ exhibit two primary peaks at 158.8 eV (4f_7/2_) and 164.1 eV (4f_5/2_), corresponding to Bi^3+^ species, along with minor peaks at 158.2 eV (4f_7/2_) and 163.5 eV (4f_5/2_) attributed to defect Bi species (Bi^*δ*+^, 0 < *δ *< 3). In comparison, O_v_‐Bi_2_O_3_ shows slightly shifted peaks assigned to Bi^3+^ at 158.9 eV (4f_7/2_) and 164.2 eV (4f_5/2_), while peaks attributed to defect Bi species appear at 158.3 eV (4f_7/2_) and 163.6 eV (4f_5/2_). The positive shift in Bi^3+^ binding energies indicates a modified electronic structure caused by disrupted Bi–O coordination due to oxygen vacancies. The removal of lattice oxygen reduces the local electron density and weakens the charge screening around Bi atoms, thereby enhancing the effective nuclear attraction and shifting the Bi 4f peaks corresponding to Bi^3+^ to higher binding energies. Notably, the ratio of defect Bi species to total Bi (sum of Bi^3+^ and defect Bi species) in O_v_‐Bi_2_O_3_ reaches 82%, significantly higher than that in I‐Bi_2_O_3_ (28%). This pronounced increase can be attributed to the charge compensation mechanism induced by oxygen vacancies formation. Upon the removal of lattice oxygen, local charge neutrality necessitates the partial reduction of Bi^3+^ to lower oxidation states (Bi^*δ*+^, 0 < *δ *< 3), thereby generating additional defect Bi species. Furthermore, XPS spectra, as shown in Figure S8, Supporting Information, were also collected to investigate the chemical states of Bi following electroreduction. The Bi 4f spectra of Bi (I‐Bi_2_O_3_) display peaks at 158.7 and 164.0 eV attributed to Bi^3+^, 158.2 and 163.5 eV corresponding to defect Bi species (Bi^*δ*+^, 0 < *δ *< 3), and 156.2 and 161.5 eV assigned to metallic Bi^0^. In comparison, Bi (O_v_‐Bi_2_O_3_) shows corresponding peaks at 159.1/164.4 eV (Bi^3+^), 158.6/163.9 eV (Bi^*δ*+^), and 156.6/161.9 eV (Bi^0^). Owing to its surface sensitivity, XPS‐detected Bi^3+^ and Bi^*δ*+^ signals can be ascribed to partial surface reoxidation caused by air exposure. The positive shift to higher binding energy in all Bi peaks for the Bi (O_v_‐Bi_2_O_3_) sample compared to the Bi (I‐Bi_2_O_3_) sample reflects a more electron‐deficient environment, which can be attributed to the residual influence of oxygen vacancies. These residual oxygen vacancies induce local lattice distortion and electronic perturbations, weakening charge screening and enhancing the effective nuclear attraction at Bi sites, thereby leading to the observed shifts in binding energy even after electroreduction. Such electronic configuration modifications resulting in more electron‐deficient Bi sites can facilitate the adsorption of the electron‐rich *OCHO intermediate, thus promoting selective formate production.

The ex situ Raman spectra were measured for the Bi_2_O_3_ precursors subjected to the electrochemical reduction with varying times to examine the effects of the oxygen vacancies on the electrochemical reduction process. As shown in Figure 2c, characteristic vibrational modes appear in the Raman spectra for I‐Bi_2_O_3_ at 68, 83, 94, 119, 139, 151, 184, 210, 279, and 313 cm^−1^, corresponding to various Bi–O stretching and bending vibrations. As the electrochemical reduction proceeds, these peaks for the characteristic vibrational modes of I‐Bi_2_O_3_ gradually diminish after 180 s, while two new small peaks emerge at 92 and 114 cm^−1^, corresponding to the *E*_g_ and *A*_1g_ vibrational modes of metallic Bi, respectively.^[^ ^29^ ^]^ It should be noted that these peaks are significantly small compared to Bi–O vibrational peaks, and the spectrum at 180 s is enlarged compared to the other spectra. In contrast, in Figure 2d, the spectra for O_v_‐Bi_2_O_3_ within 60 s exhibit the peaks at 66, 81, 92, 117, 137, 149, 182, 208, 277, and 311 cm^−1^, whose Raman shifts are smaller than those observed in the I‐Bi_2_O_3_ spectra. This peak shift indicates weaker Bi—O bonds in O_v_‐Bi_2_O_3_, which is attributed to the lattice distortions caused by oxygen vacancies. Specifically, the introduction of oxygen vacancies in Bi_2_O_3_ disrupts the local coordination environment of Bi atoms, leading to the formation of undercoordinated Bi sites with reduced Bi—O coordination numbers. The resulting local structural distortion weakens the Bi—O bond strength due to the removal of bridging oxygen atoms that originally stabilized the Bi–O framework. As evidenced by the rapid disappearance of the Raman peaks for Bi_2_O_3_ after 120 s, the electrochemical reduction process was significantly accelerated in the presence of oxygen vacancies, which is also concomitant with the emergence of metallic Bi peaks with smaller Raman shifts at 90 and 112 cm^−1^. The accelerated transformation can be attributed to the presence of oxygen vacancies accompanied by Bi defects, which enhance charge transport and create undercoordinated Bi sites with weakened Bi—O bonds. This local structural distortion reduces the thermodynamic stability of Bi^3+^, thereby lowering the energy barrier for its reduction and facilitating the rapid formation of metallic Bi.

### 2.2 Electrochemical Properties

**Figure 3:**
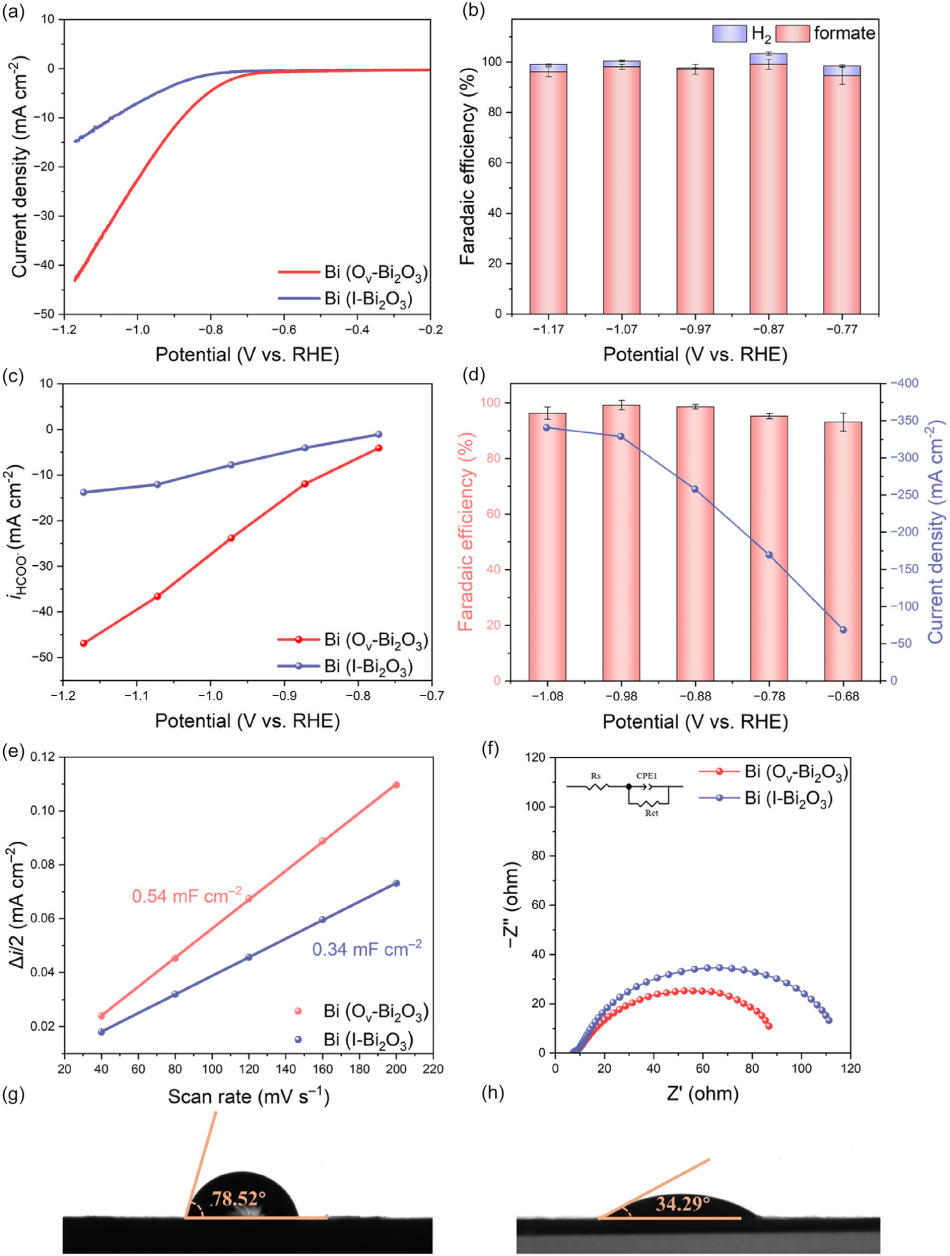
a) LSVs of Bi (O_v_‐Bi_2_O_3_) and Bi (I‐Bi_2_O_3_) in 0.2 M CO_2_‐saturated KHCO_3_. b) FEs of ERCO_2_ products of Bi (O_v_‐Bi_2_O_3_). c) Partial current density for formate production of Bi (O_v_‐Bi_2_O_3_) and Bi (I‐Bi_2_O_3_) at different potentials in H‐type cell. d) FE_formate_ and current density at different potentials of Bi (O_v_‐Bi_2_O_3_) in the flow cell. e) The average current density differences versus scan rate at –0.27 V versus RHE of Bi (O_v_‐Bi_2_O_3_) and Bi (I‐Bi_2_O_3_). f) Nyquist plots measured for Bi (O_v_‐Bi_2_O_3_), Bi (I‐Bi_2_O_3_) in the frequency range from 0.1 to 10^5^ Hz at OCP with a voltage amplitude of 5 mV. The equivalent circuit used to obtain *R*_ct_ values is also shown. Contact angles of g) Bi (O_v_‐Bi_2_O_3_) and h) Bi (I‐Bi_2_O_3_) with deionized water.

The ERCO_2_ performance of Bi (O_v_‐Bi_2_O_3_) and Bi (I‐Bi_2_O_3_) was initially evaluated in an H‐type cell (Figure S9, Supporting Information) using 0.2 M KHCO_3_ aqueous solution as the electrolyte. As shown in Figure S10, Supporting Information, linear sweep voltammograms (LSVs) measured in N_2_‐saturated 0.2 M KHCO_3_ aqueous solution revealed that Bi (O_v_‐Bi_2_O_3_) exhibited a significantly lower current density compared to Bi (I‐Bi_2_O_3_). Since CO_2_ was absent in this environment, the observed current density mainly originated from the competing hydrogen evolution reaction (HER). The remarkably suppressed lower current density on Bi (O_v_‐Bi_2_O_3_) exhibited that the introduction of small‐angle grain boundaries effectively inhibits HER. In contrast, as shown in **Figure** **3**a, under CO_2_‐saturated conditions, LSVs showed a significant enhancement in the ERCO_2_ activity for Bi (O_v_‐Bi_2_O_3_) compared to that for Bi (I‐Bi_2_O_3_). Notably, Bi (O_v_‐Bi_2_O_3_) exhibited a higher onset potential (i.e., lower overpotential) and a steeper increase in current density, suggesting a lower energy barrier for initiating CO_2_ reduction, a larger density of active sites, and enhanced binding of reaction intermediates.^[^ ^30^ ^]^ To further evaluate the catalytic performance, chronoamperometry (controlled potential electrolysis) was conducted within a potential range of −0.77 to −1.17 V versus RHE for 30 min (Figure S11, Supporting Information). The gas products were examined by online gas chromatography, and the obtained liquid products were evaluated via high‐performance liquid chromatography (HPLC) using an external standard method (Figure S12, Supporting Information). Bi (O_v_‐Bi_2_O_3_) exhibited an outstanding formate selectivity (Figure 3b), maintaining FE_formate_ above 90% across the entire testing range and approaching 100% most at −1.07 V versus RHE, outperforming most Bi‐based catalysts previously reported (Table S1, Supporting Information). In comparison, the Bi (I‐Bi_2_O_3_) catalyst only reached a maximum FE_formate_ of 83.1% at −0.87 V versus RHE (Figure S13, Supporting Information). Moreover, as shown in Figure 3c, the formate partial current density for Bi (O_v_‐Bi_2_O_3_) is significantly higher than that of Bi (I‐Bi_2_O_3_), based on the calculated data of FE_formate_. The enhanced formate selectivity is likely attributed to the strengthened CO_2_ adsorption on the Bi defects and the suppression of H_2_ formation at small‐angle grain boundaries in Bi (O_v_‐Bi_2_O_3_).^[^ ^31^ ^]^ Gas analysis shown in Figure 3b and S13, Supporting Information, revealed that Bi (O_v_‐Bi_2_O_3_) exclusively produces H_2_ as a gas product, whereas Bi (I‐Bi_2_O_3_) generates both H_2_ and CO gases during ERCO_2_. This observation implies that oxygen vacancy‐driven structural reconstruction modulates the local electronic environment of Bi active sites, thereby favoring the adsorption and stabilization of *OCHO intermediates and promoting selective formate production. Furthermore, to overcome the CO_2_ mass transfer limitation caused by its low solubility in aqueous electrolyte within a conventional H‐type cell, a flow cell system (Figure S14, Supporting Information) was employed. In this system, 1 M KOH solution was used as the electrolyte to provide high ionic conductivity and support high current operation for industrial applications, while a gas diffusion electrode (GDE) was incorporated to ensure efficient CO_2_ delivery. This setup allows for a more accurate evaluation of the electrocatalytic performance of Bi (O_v_‐Bi_2_O_3_) under practical conditions for ERCO_2_. As depicted in Figure S15 and S16, Supporting Information, CO_2_ was directly delivered through the diffusion layer to the catalyst surface, thereby ensuring improved mass transport and accelerated reaction kinetics. Benefiting from the enhanced mass transport environment, the Bi (O_v_‐Bi_2_O_3_) catalyst achieved a current density of 340 mA cm^−2^ at −1.08 V versus RHE with FE_formate_ of 96% (Figure 3d), exceeding the industrial benchmark of 200 mA cm^−2^ and highlighting its potential for industrial‐scale ERCO_2_ applications.^[^ ^32^ ^]^ Notably, the corresponding formate formation rate achieved 6.09 mmol h^−1^ cm^−2^ at the potential of −1.08 V versus RHE, reflecting not only excellent catalytic activity but also remarkable product generation efficiency under industrially applicable conditions. Across a wide potential range from –0.88 to −1.08 V versus RHE, Bi (O_v_‐Bi_2_O_3_) maintained high FE_formate_ exceeding 95% and current density above 200 mA cm^−2^, outperforming most previously reported Bi‐based electrocatalysts (Table S2, Supporting Information). To investigate the electrochemical properties of Bi (O_v_‐Bi_2_O_3_) and Bi (I‐Bi_2_O_3_) catalysts, cyclic voltammograms (CVs) and electrochemical impedance spectroscopy (EIS) were conducted. By analyzing CVs at various scan rates, the double‐layer capacitance (*C*_dl_) values were calculated as 0.54 mF cm^−2^ for Bi (O_v_‐Bi_2_O_3_) and 0.34 mF cm^−2^ for Bi (I‐Bi_2_O_3_), respectively, based on the linear relationship between capacitive current density and scan rates (Figure 3e and S17, Supporting Information). The higher *C*_dl_ of Bi (O_v_‐Bi_2_O_3_) compared to Bi (I‐Bi_2_O_3_) suggests a greater electrochemical active surface area (ECSA), which is proportional to *C*_dl_, indicating more efficient utilization of active sites.^[^ ^33^ ^]^ EIS measurements were performed to evaluate the electron transfer capability of the catalyst; the Nyquist plots fitted with an equivalent circuit model are depicted in Figure 3f and S18, Supporting Information. The charge transfer resistance (*R*_ct_) of Bi (O_v_‐Bi_2_O_3_) was determined to be 94.4 Ω, significantly lower than that of Bi (I‐Bi_2_O_3_) (115.1 Ω). The smaller *R*_ct_ reflects improved catalytic kinetics in Bi (O_v_‐Bi_2_O_3_), enhancing electron transfer efficiency and reducing charge‐transfer resistance.^[^ ^34^, ^35^ ^]^ This accelerated electron transfer promotes rapid electrochemical reactions, facilitating the initial electron transfer step to convert adsorbed CO_2_ molecules into *CO_2_^**−**^ intermediates.^[^ ^36^, ^37^ ^]^ Moreover, as depicted in Figure 3g,h, the contact angle of Bi (I‐Bi_2_O_3_) was measured to be 34.29°, while Bi (O_v_‐Bi_2_O_3_) exhibited a significantly higher contact angle of 78.52°, indicating a higher hydrophobicity. This mild hydrophobicity may enhance CO_2_ adsorption by reducing excessive water coverage at the catalyst–electrolyte interface, while simultaneously suppressing the competing HER.

**Figure 4:**
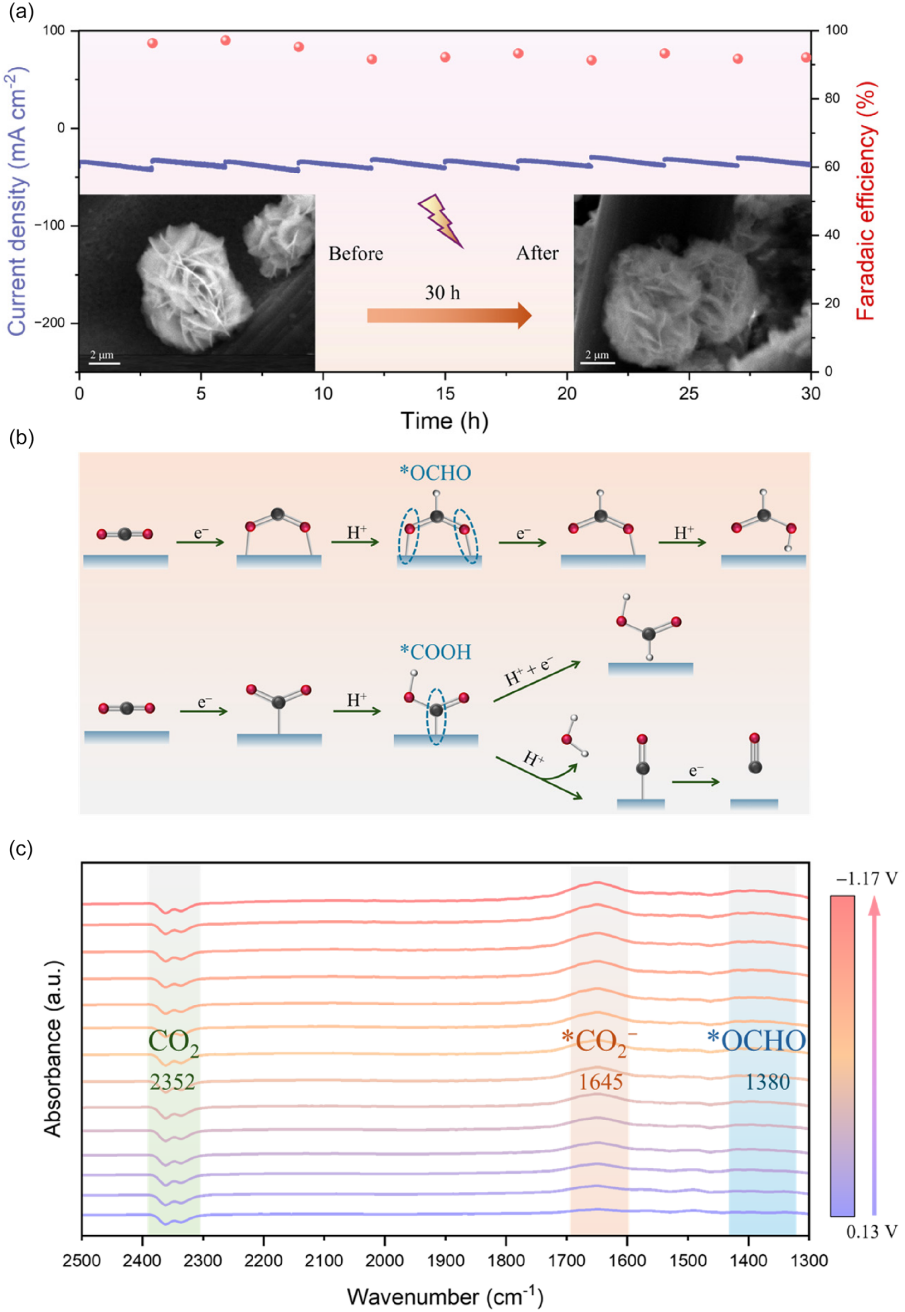
a) The FE_formate_ and current density of Bi (O_v_‐Bi_2_O_3_) during long‐term ERCO_2_ for 30 h in H‐type cell at −1.07 V versus RHE. b) Schematic diagram of possible reaction pathways of ERCO_2_ to formate through the formation of different intermediates. c) In situ ATR‐SEIRAS spectra of Bi (O_v_‐Bi_2_O_3_) catalyst from 0.13 to –1.17 V versus RHE.

The stability of Bi (O_v_‐Bi_2_O_3_) was evaluated in the H‐type cell at a constant potential of −1.07 V versus RHE, with the formate concentration measured after each 3 h electrolysis period. The electrolyte was replaced every 3 h to prevent excessive HCOO^**−**^ coverage at the electrode, which could otherwise affect the current density.^[^ ^38^ ^]^ The test was therefore conducted under extended electrolysis with intermittent electrolyte refilling. After 30 h of continuous operation, the FE_formate_ slightly dropped but still stayed above 90%, demonstrating the excellent stability of the Bi (O_v_‐Bi_2_O_3_) catalyst (**Figure** **4**a). XRD and SEM were employed to examine potential changes in phase structure and morphology of the catalyst after 30 h of continuous electrolysis (Figure S19 and S20, Supporting Information). The XRD pattern exhibited no discernible changes, and SEM images confirmed that the catalyst retained its highly hierarchical sheet‐like morphology without any structural collapse, providing further evidence of its excellent stability. Nevertheless, the Bi (I‐Bi_2_O_3_) catalyst failed to maintain stable performance, with its FE_formate_ dropping below 40% after 6 h of electrochemical reduction (Figure S21, Supporting Information).

### 2.3 Mechanism Analysis

The adsorption experiment was performed by measuring LSVs in 0.1 M KOH, with OH^−^ used as substitute ions to evaluate the binding capability of Bi (I‐Bi_2_O_3_) and Bi (O_v_‐Bi_2_O_3_) toward *CO_2_^**−**^ intermediates.^[^ ^39^, ^40^ ^]^ The lower OH^−^ adsorption potential observed for Bi (O_v_‐Bi_2_O_3_) (Figure S22, Supporting Information) reflects its stronger binding affinity for *CO_2_^**−**^. This enhanced affinity can be attributed to the abundant small grain boundaries and defects in Bi (O_v_‐Bi_2_O_3_), which offer additional active sites and create localized electronic conditions that favor *CO_2_^**−**^ binding. As illustrated in the proposed reaction pathways (Figure 4b), *CO_2_^**−**^ can be further protonated to form either the *OCHO or *COOH intermediate.^[^ ^17^, ^41^, ^42^ ^]^ The *OCHO intermediate typically exclusively leads to formate, whereas *COOH intermediate may subsequently yield either formate or CO. Therefore, to gain deeper insights into the electrocatalytic mechanism and reaction pathways of ERCO_2_ on Bi (O_v_‐Bi_2_O_3_), in situ ATR‐SEIRAS was performed in CO_2_‐saturated 0.2 M KHCO_3_ solution over a potential range from 0.13 V versus RHE to –1.17 V vs. RHE (Figure 4c). The peak at 2352 cm^−1^ is attributed to adsorbed CO_2_ molecules, while the band observed at ≈1645 cm^−1^ arises from surface‐adsorbed *CO_2_^**−**^ intermediate species, formed upon exposure of Bi (O_v_‐Bi_2_O_3_) to the CO_2_/H_2_O environment.^[^ ^43^, ^44^ ^]^ The intensity of the characteristic peaks of *OCHO at 1380 cm^−1^ gradually increases with lowering the applied potential. This enhancement can be attributed to the vibration of the O—C—O bond in the two‐oxygen bridge‐bonded *OCHO species.^[^ ^45^ ^]^ The absence of a peak in the 1900–2100 cm^−1^ range, corresponding to *CO intermediates, suggests that CO production on Bi (O_v_‐Bi_2_O_3_) is significantly suppressed, further supported by the absence of CO formation during the ERCO_2_ as previously shown in Figure 3b.^[^ ^46^ ^]^ Consequently, the formation of HCOOH on Bi (O_v_‐Bi_2_O_3_) predominantly follows the CO_2_ → *CO_2_^**−**^ → *OCHO → HCOOH pathway rather than the CO_2_ → *CO_2_^**−**^ → *COOH → HCOOH route, thereby offering a mechanistic rationale for the observed high selectivity toward formate.

## 3 Conclusion

In this study, we demonstrated a novel structural transformation pathway from Bi_5_O_7_NO_3_ to oxygen vacancy‐rich Bi_2_O_3_ with unique morphological features, followed by electrochemical reconstruction to form defect‐rich Bi nanoflowers for highly selective ERCO_2_ to formate above 90% for more than 30 h. The strong alkaline treatment of Bi_5_O_7_NO_3_ triggered structural evolution and defect formation, which critically facilitated extensive surface reconstruction during electrochemical reconfiguration, thereby maximizing active site exposure and enhancing the adsorption and stabilization of *OCHO intermediates in the resulting Bi catalyst with defects and small‐angle grain boundaries. This study demonstrates that synthesis‐dependent variations in the density of small‐angle grain boundaries and structural defects profoundly affect the ERCO_2_ pathway and Faradaic efficiency. Building on these findings, this work provides a novel and efficient strategy for designing high‐performance electrocatalysts by leveraging structural transformation, defect engineering, and surface reconstruction, offering valuable insights for advancing CO_2_ reduction technologies and contributing to the development of sustainable energy solutions.

## 4 Experimental Section

4.1

4.1.1

##### Materials

Bismuth nitrate pentahydrate (Bi(NO_3_)_3_·5H_2_O, 99.5%) was purchased from Nacalai Tesque, Inc. Nitric acid (HNO_3_, 60.0%), ethanol (C_2_H_5_OH, 99.5%), bismuth oxide (Bi_2_O_3_, 99.9%), isopropyl alcohol ((CH_3_)_2_CHOH, 99.7%), Nafion solution (5 wt%), and potassium bicarbonate (KHCO_3_, 99.5%) were obtained from Fujifilm Wako Pure Chemical Corporation. All materials were used as received without further purification.

##### Preparation of Catalysts and Electrodes

###### Preparation of Catalysts and Electrodes: Synthesis of Catalyst Precursors (Bi_5_O_7_NO_3_)

To synthesize Bi_5_O_7_NO_3_, 480 mg of Bi(NO_3_)_3_·5H_2_O was placed in a crucible, followed by 20 and 4 mL of C_2_H_5_OH and HNO_3_, respectively. The solution was ultrasonicated for 30 min and then stirred at room temperature for 3 h. Afterward, the mixture was dried on a heating plate at 80 °C, and the resulting solid was calcined in a muffle furnace at 400 °C or 5 h to obtain Bi_5_O_7_NO_3_. For the synthesis of O_v_‐Bi_2_O_3_, the as‐prepared Bi_5_O_7_NO_3_ was dispersed in 20 mL of 6 M KOH aqueous solution and stirred at room temperature for 5 min. The resulting precipitate was collected using centrifugation and thoroughly washed thrice with deionized water and C_2_H_5_OH.

##### Preparation of Catalysts and Electrodes: Electrode Preparation of Bi (O_v_‐Bi_2_O_3_) Catalyst

The working electrode was fabricated using carbon paper (1 cm × 1 cm) as the substrate, which underwent sequential cleaning with deionized water and C_2_H_5_OH. For catalyst ink preparation, precisely 1 mg of the O_v_‐Bi_2_O_3_ material was mixed with 400 μL isopropyl alcohol (C_3_H_8_O) and 10 μL of 5 wt% Nafion solution in a glass vial. Following 30 min of ultrasonication to achieve a homogeneous dispersion, the resulting suspension was uniformly dropped onto the pretreated carbon paper using a pipette gun. Electrochemical reduction was then subsequently performed at a constant potential of −1.07 V versus RHE for 1 h to generate the Bi (O_v_‐Bi_2_O_3_) catalyst.

##### Preparation of Catalysts and Electrodes: Electrode Preparation of Bi (I‐Bi_2_O_3_) Catalyst

Commercially available Bi_2_O_3_ with an intact phase structure was denoted as I‐Bi_2_O_3_. The synthesis process of the Bi (I‐Bi_2_O_3_) catalyst followed a procedure similar to that of the Bi (O_v_‐Bi_2_O_3_) catalyst, except that I‐Bi_2_O_3_ was used to prepare the catalyst ink.

##### Material Characterizations

The crystal structures were analyzed using XRD (Ultima IV, Rigaku Co., Ltd.) with a molybdenum X‐ray tube (*λ* = 0.7107 Å). SEM and TEM, respectively images were obtained using a JEOL JSM‐6010 LA SEM and JEOL JEM‐F200 transmission electron microscope equipped with EDS, respectively, to observe the microscopic morphology of the materials. XPS was conducted using a ULVAC‐PHI Quantera Instrument to evaluate the electronic states of the elements on the catalyst surface. The adventitious carbon C 1s peak was calibrated to 284.4 eV, and all spectra were fitted using a Gauss–Lorentz product line shape with a Shirley background. Raman spectra were acquired using a WITec alpha300R spectrometer with a 532 nm laser, and contact angle measurements were conducted using a JY‐82C video contact angle analyzer at room temperature. EPR measurements were performed using a Bruker EMXplus‐6/1 spectrometer at room temperature. In situ ATR‐SEIRAS was performed using a Thermo Fisher Scientific Nicolet iS50 spectrometer.

##### Electrochemical Measurements

All the electrochemical measurements were conducted at room temperature. Initially, H‐type cell was employed for electrolysis, utilizing 0.2 M KHCO_3_ as the electrolyte, with the cathode and anode compartments separated by a Nafion117 ion exchange membrane. A three‐electrode configuration was adopted, comprising the modified carbon paper electrode as the working electrode, a Pt mesh (2.5 cm × 3.5 cm) as the counter electrode, and an Ag/AgCl electrode immersed in a saturated KCl aqueous solution as the reference electrode. The measured potentials versus the Ag/AgCl electrode were converted to the RHE scale using the following equation: $E_{\text{RHE}}=E_{\text{Ag/AgCl}}+0.197+0.0591\;\text{pH}$. The electrochemical double‐layer capacitance was determined to evaluate the ECSA through CVs at various scan rates (40, 80, 120, 160, and 200 mV s^−1^) within a potential range from −0.17 to −0.37 V versus RHE. EIS was conducted within a frequency range from 10^5^ to 0.1 Hz at open‐circuit potential (OCP), with an applied voltage amplitude of 5 mV.

To further evaluate the electrochemical performance of the catalysts, a flow cell configuration was employed. The flow cell comprised gas, catholyte, and anolyte chambers, each equipped with an inlet and outlet to facilitate CO_2_ supply and electrolyte circulation. Both the anolyte and catholyte were 1 M KOH solutions separated by an anion exchange membrane, and the CO_2_ flow rate was maintained at 30 mL min^−1^ using an electronic flowmeter. The GDE, platinum foil, and Ag/AgCl electrode were used as the working, counter, and reference electrodes, respectively. The exposed areas of the cathode, anode, and membrane window were all 1 cm^2^.

##### Product Analysis

The concentration of dissolved products in the electrolyte was determined by HPLC (Shimadzu Nexera LC‐40D), while the gas products were analyzed by gas chromatography using an Agilent 490 Micro GC. The FE_formate_ was calculated using the following formula.

(1) $$\text{FE}\;(\%)=\frac{z\cdot n\cdot F}{Q}$$

where *n* represents the moles of the product, *F* is the Faraday constant (96 485 C mol^−1^), *Q* is the total electronic charge transferred during electrolysis, and *z* is 2, which is the number of moles of electrons transferred to form 1 mole of formate.

The FEs of CO and H_2_ were calculated as follows

(2) $${\text{FE}}_{H_{2}/\text{CO}}=\frac{\frac{v}{60\;s\;\min^{-1}}\times \frac{c}{22400\;{\text{cm}}^{3}\;{\text{mol}}^{-1}}\times N\times F}{i_{\text{total}}}$$

where *N* is the mole number of electrons transferred to form 1 mole of the corresponding gas product, *v* is the CO_2_ flow rate (30 mL min^−1^), *c* is the concentration of the gas products, and *i* is the current density.

## Conflict of Interest

The authors declare no conflict of interest.

## Supporting information

Supplementary Material

## Data Availability

The data that support the findings of this study are available from the corresponding author upon reasonable request.
