# Supplementary material for: Defect‐Driven Reconstruction of Bismuth Nanoflowers via Precursor Engineering for Highly Efficient CO2‐to‐Formate Electrochemical Reduction
Source: Small Sci. 2025 Aug 20;5(10):2500296. doi: 10.1002/smsc.202500296 (PMC12499387; doi:10.1002/smsc.202500296)
Supplement: Supplementary file 1 — Supplementary Material [file SMSC-5-2500296-s001.pdf]

Supporting Information

Defect-Driven Reconstruction of Bismuth Nanoflowers via Precursor Engineering for Highly Efficient CO_2-to-Formate Electroreduction

Jiaying Yan[a], Masao Kamiko[a], Teruyasu Mizoguchi[a], Shunsuke Yagi[a,]*

[a] Institute of Industrial Science, The University of Tokyo, 4-6-1 Komaba, Meguro-ku, Tokyo 153-8505, Japan

*Corresponding author: E-mail: syagi@iis.u-tokyo.ac.jp (S. Yagi)

Content

Figure S1. Photograph of as-synthesized (a) $Bi_5O_7NO_3$, (b)O_v-Bi_2O_3 and (c) Bi (O_v-Bi_2O_3) powders.

Figure S2. (a) HAADF-STEM image and corresponding EDS elemental mapping, and (b) HRTEM image of $Bi_5O_7NO_3$.

Figure S3. (a) HRTEM image of O_v-Bi_2O_3, (b) HAADF-STEM image and corresponding EDS elemental maps of (c) Bi and (d) O.

Figure S4. (a) XRD pattern and (b,c) SEM images of I-Bi_2O_3.

Figure S5. HRTEM images of Bi (O_v-Bi_2O_3).

Figure S6. (a) XRD pattern and (b,c) SEM images of Bi (I-Bi_2O_3).

Figure S7. Fitted EPR spectra of O_v-Bi_2O_3 and I-Bi_2O_3.

Figure S8. High-resolution XPS spectra at Bi 4f regions of Bi (O_v-Bi_2O_3) and Bi (I-Bi_2O_3).

Figure S9. Photograph of H-type cell for $ERCO_2$.

Figure S10. LSVs of Bi (O_v-Bi_2O_3) and Bi (I-Bi_2O_3) in 0.2 M N_2-saturated $KHCO_3$.

Figure S11. Chronoamperograms of (a) Bi (O_v-Bi_2O_3) and (b) Bi (I-Bi_2O_3) in CO_2-saturated 0.2 M $KHCO_3$ electrolyte at different potentials.

Figure S12. Linear relationship between the known concentrations of $HCOO^-$ and the relative peak area measured by high-performance liquid chromatography.

Figure S13. Faradaic efficiencies of $ERCO_2$ products of Bi (I-Bi_2O_3).

Figure S14. Diagram of the gas diffusion electrode (GDE) configuration for $ERCO_2$.

Figure S15. Photograph of flow cell for $ERCO_2$.

Figure S16. Installation diagram of flow cell for $ERCO_2$.

Figure S17. CVs of the catalysts at different sweep speeds: (a) Bi (O_v-Bi_2O_3) and (b) Bi (I-Bi_2O_3).

Figure S18. Nyquist plots of (a) Bi (O_v-Bi_2O_3) and (b) Bi (I-Bi_2O_3) (solid symbols are experimental results and solid lines are fitted curves).

Figure S19. XRD pattern of (a) Bi (O_v-Bi_2O_3) after stability test for 30 h, (b) pristine carbon paper.

Figure S20. SEM image of Bi (O_v-Bi_2O_3) after stability test for 30 h at –1.07 V vs. RHE in H-type cell with 0.2 M $KHCO_3$ electrolyte.

Figure S21. $FE_{formate}$ and current density of Bi (I-Bi_2O_3) during long-term CO_2 electroreduction at –1.07 V vs. RHE for 6 h in H-type cell with 0.2 M $KHCO_3$ electrolyte.

Figure S22. Oxidative LSVs measured in 0.1 M KOH on Bi (O_v-Bi_2O_3) and (b) Bi (I-Bi_2O_3) at scan rate of 0.05 V s^{-1}.

Table S1. Comparison of various Bi-based electrocatalysts for $ERCO_2$ to formate conducted in H-type cell.

Table S2. Comparison of reported Bi-based catalysts for formate production by $ERCO_2$ in 1 M KOH in flow cell.

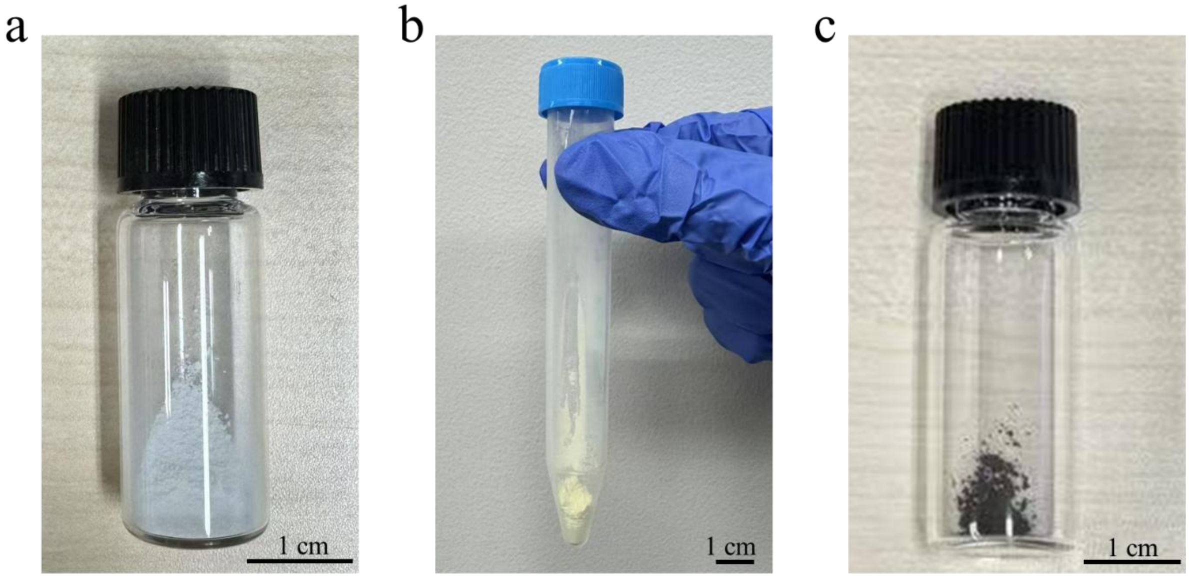

Figure S1. Photograph of as-synthesized (a) $Bi_5O_7NO_3$, (b)O_v-Bi_2O_3 and (c) Bi (O_v-Bi_2O_3) powders.

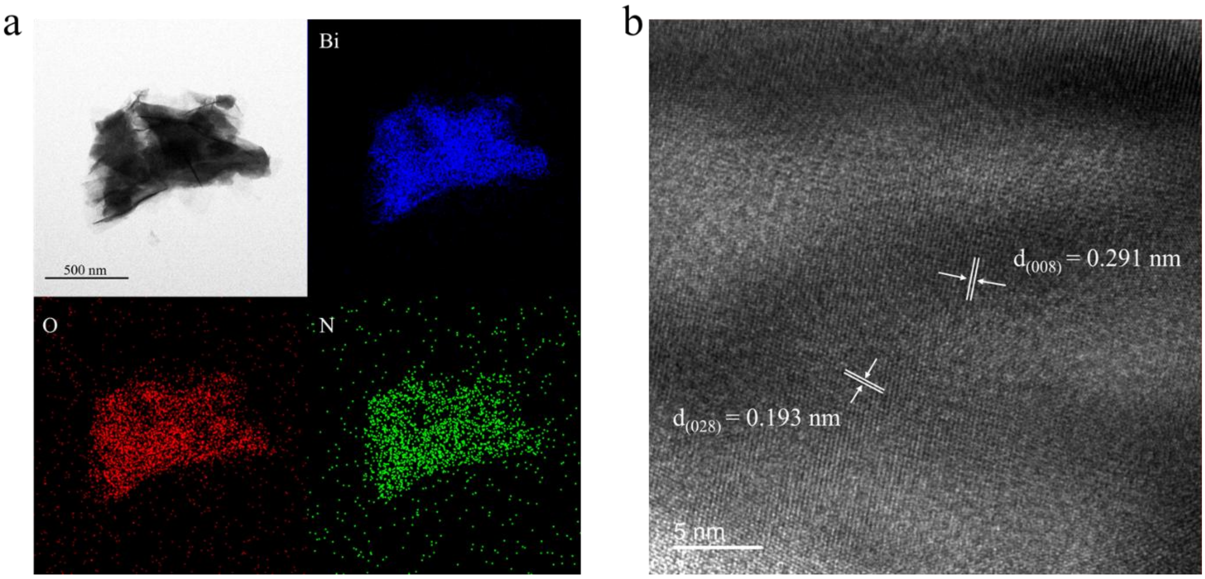

Figure S2. (a) HAADF-STEM image and corresponding EDS elemental mapping, and (b) HRTEM image of $Bi_5O_7NO_3$.

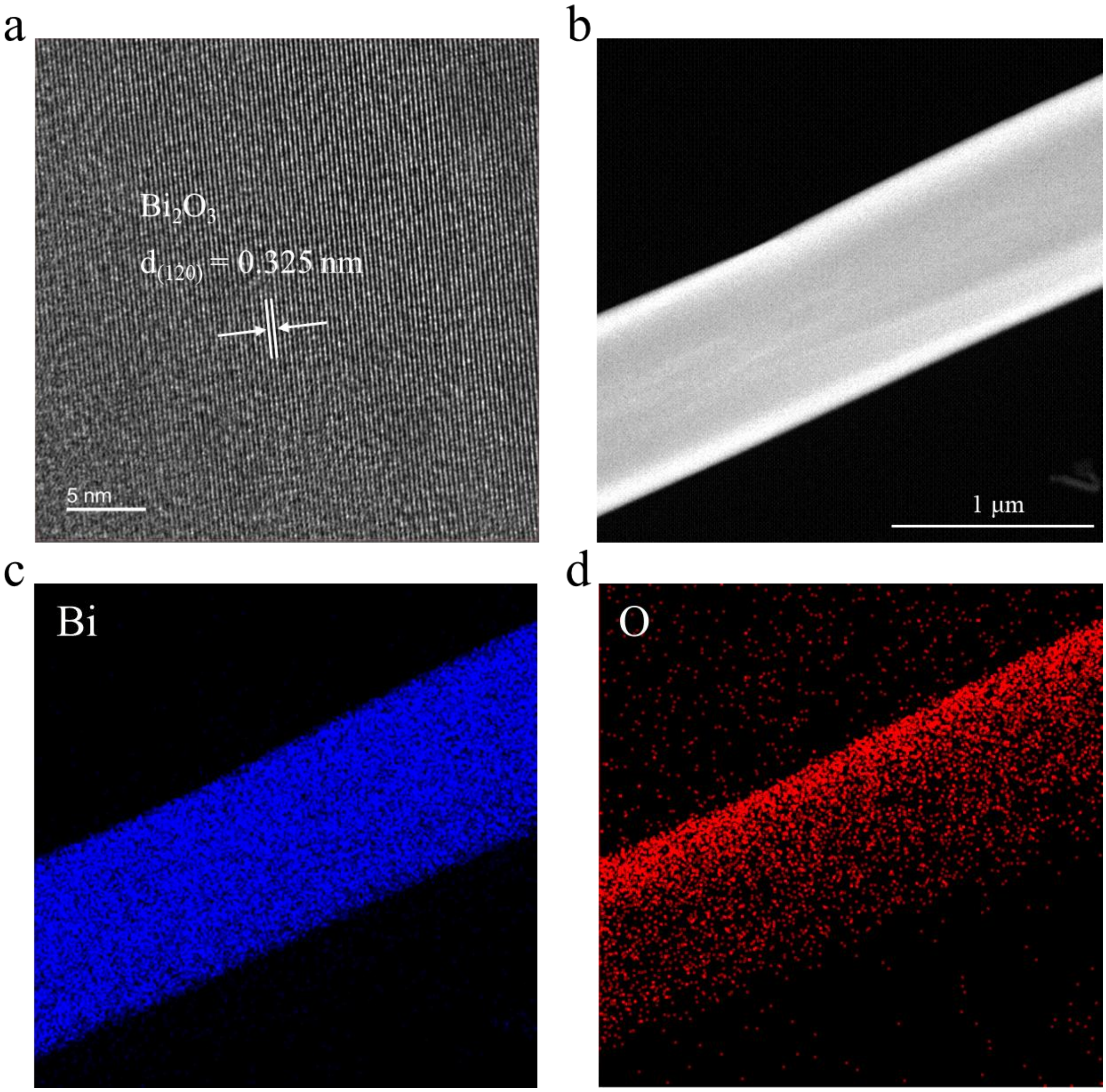

Figure S3. (a) HRTEM image of O_v-Bi_2O_3, (b) HAADF-STEM image and corresponding EDS elemental maps of (c) Bi and (d) O.

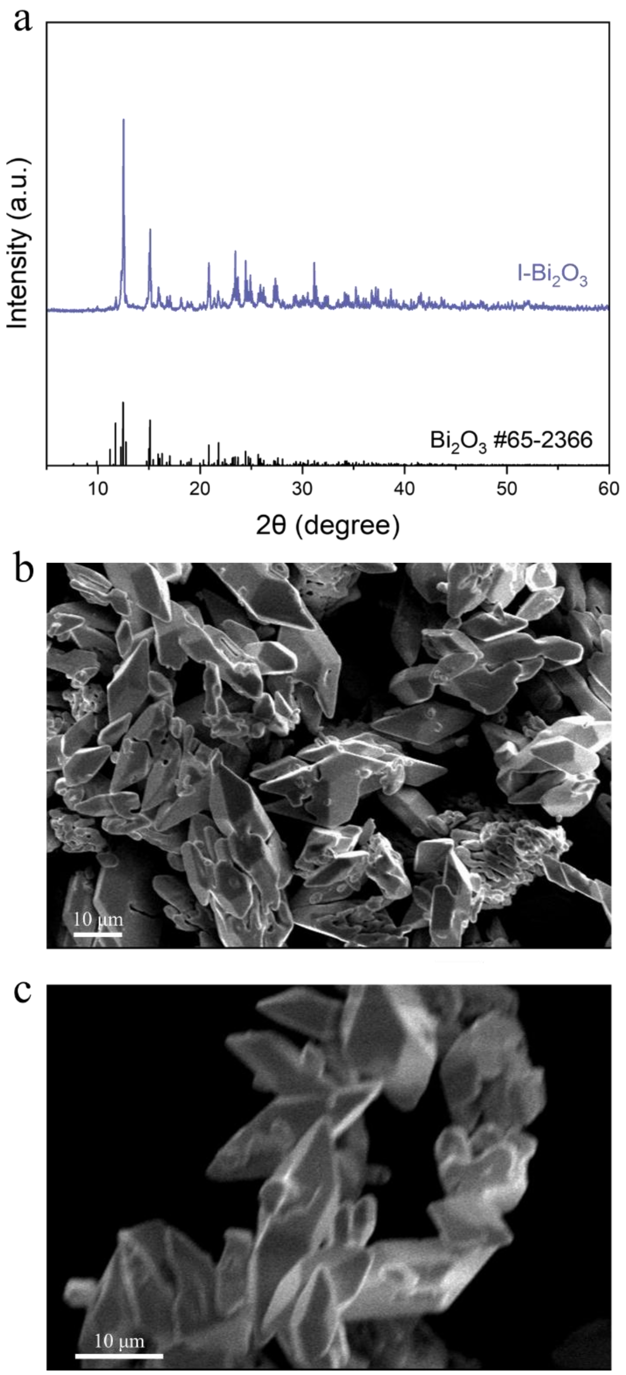

Figure S4. (a) XRD pattern measured with Mo $K\alpha$ radiation and (b,c) SEM images of I-Bi_2O_3.

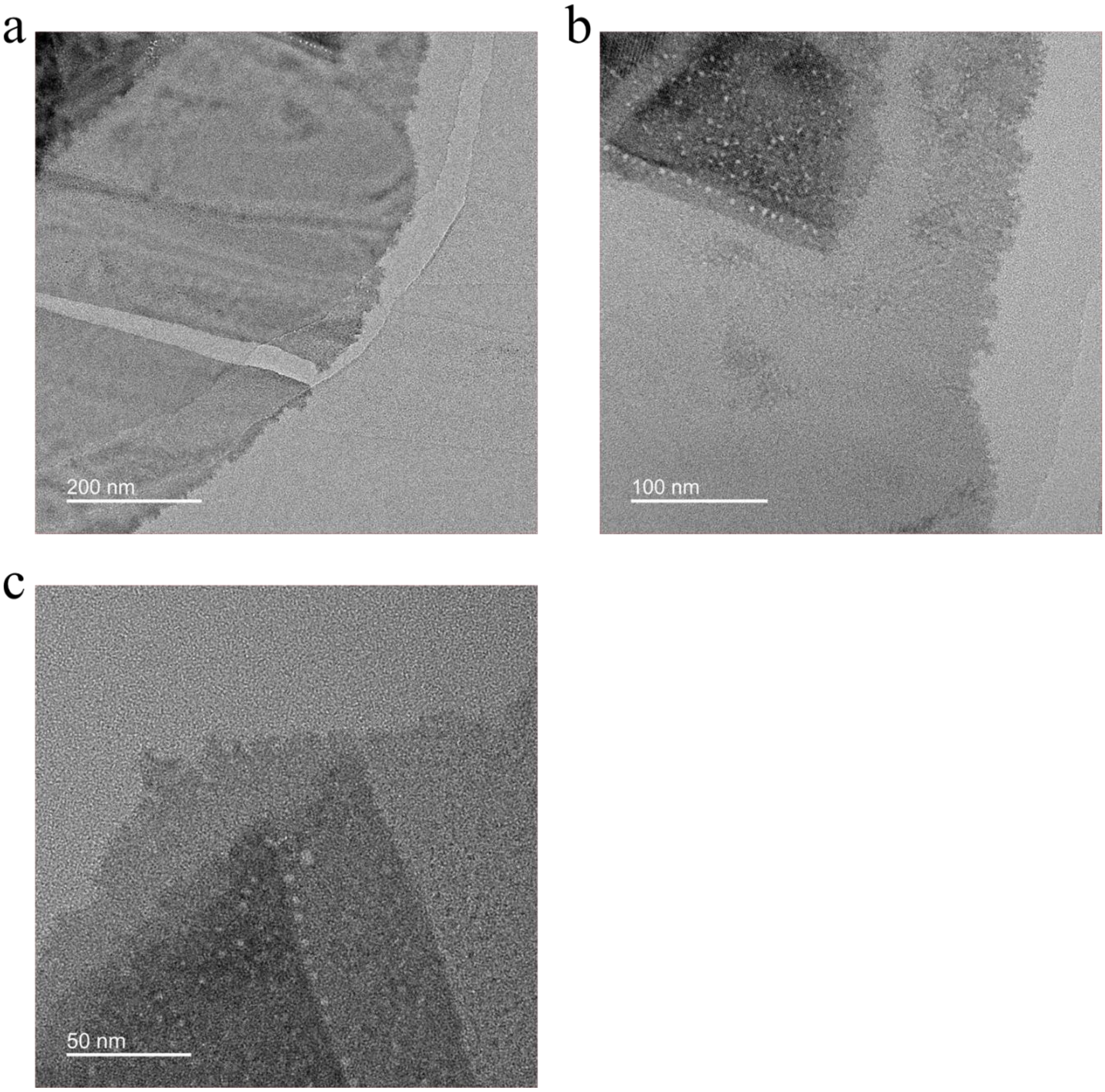

Figure S5. HRTEM images of Bi (O_V-Bi_2O_3).

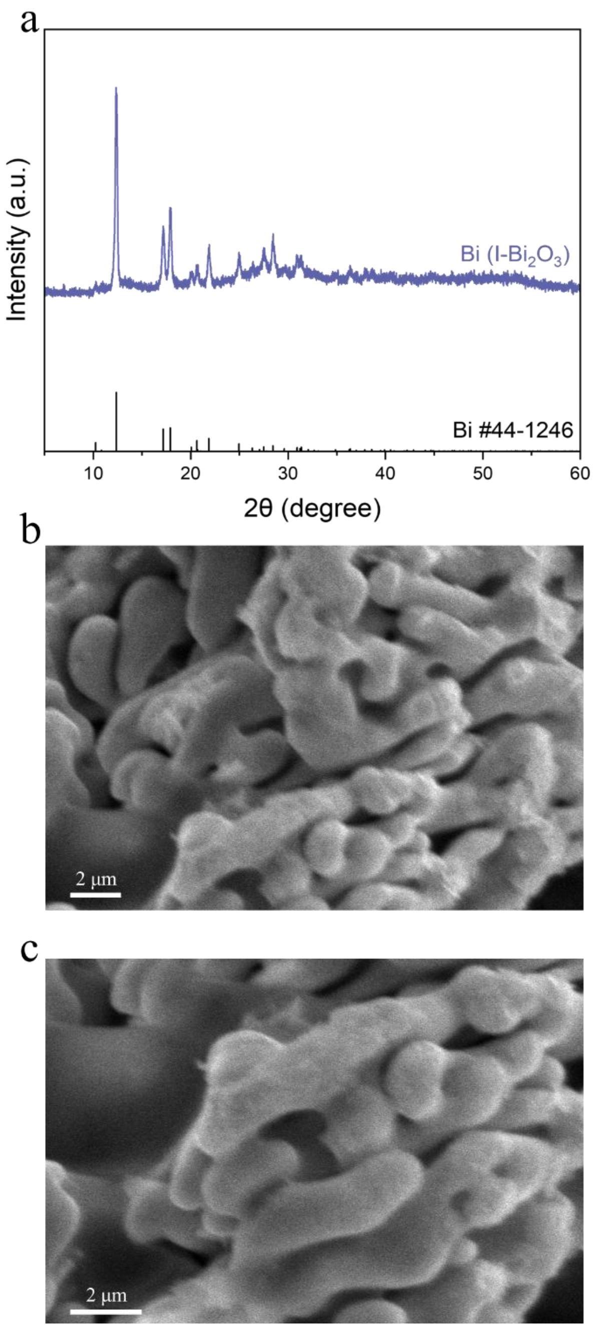

Figure S6. (a) XRD pattern measured with Mo *K*α radiation and (b,c) SEM images of Bi (I-Bi_2O_3).

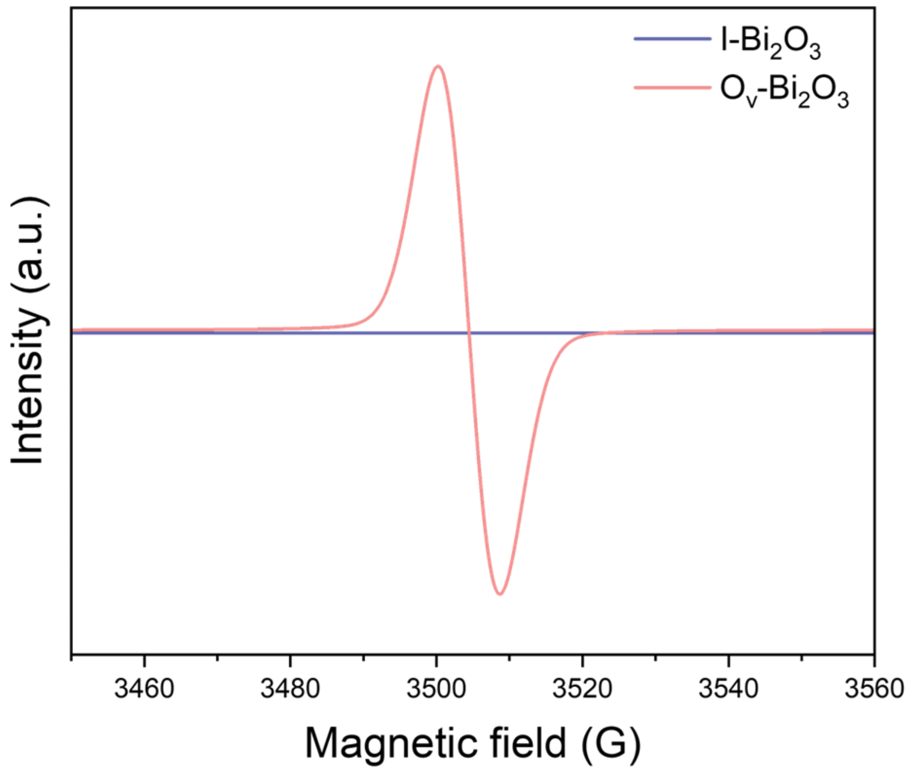

Figure S7. Fitted EPR spectra of O_v-Bi_2O_3 and I-Bi_2O_3.

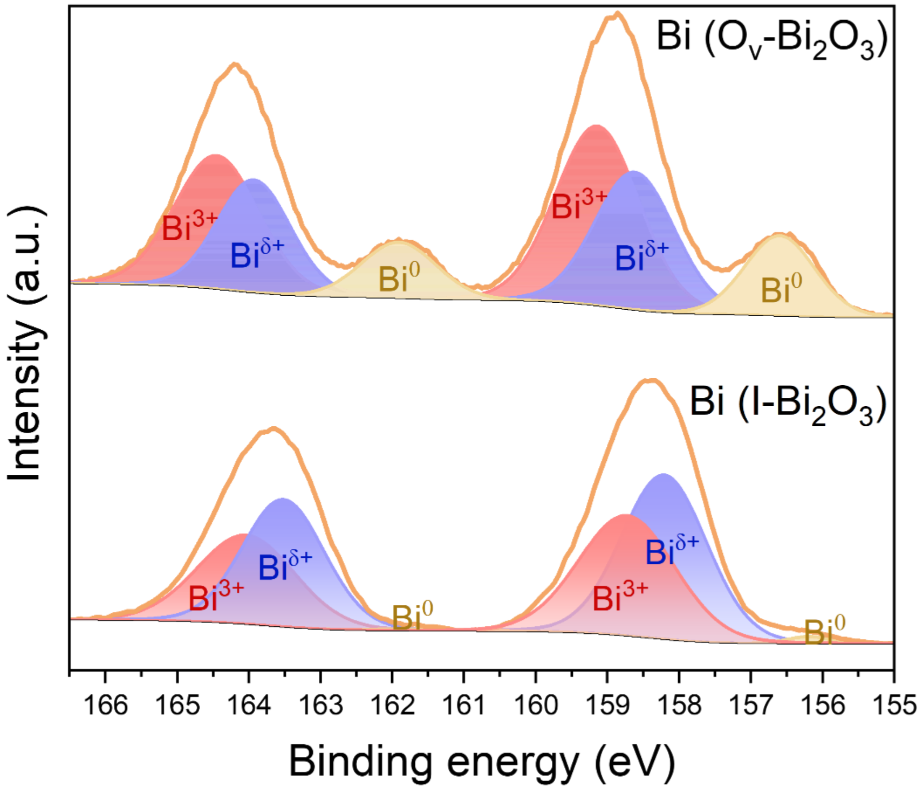

Figure S8. High-resolution XPS spectra at Bi 4f regions of Bi (O_v-Bi_2O_3) and Bi (I-Bi_2O_3).

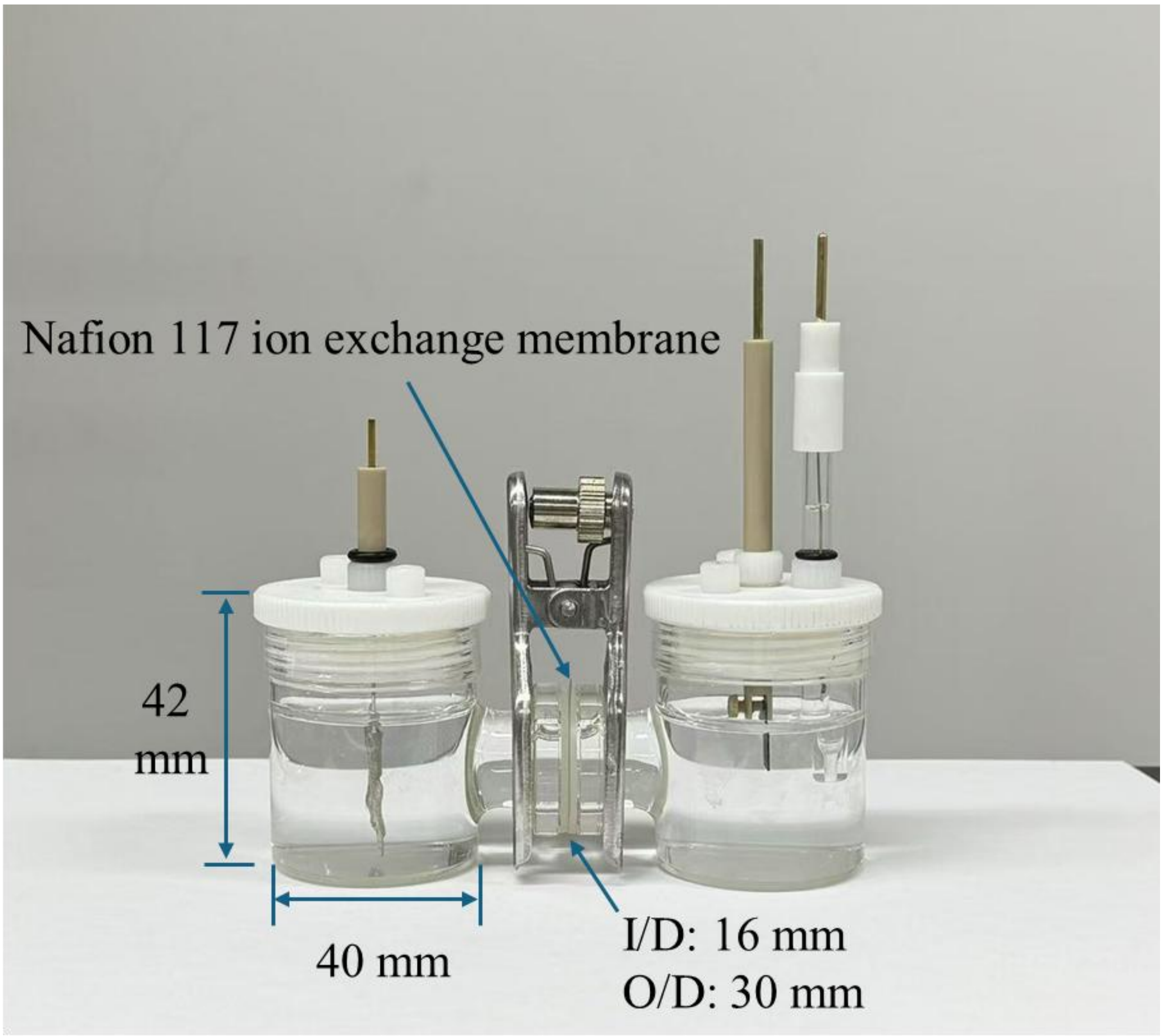

Figure S9. Photograph of H-type cell for $ERCO_2$.

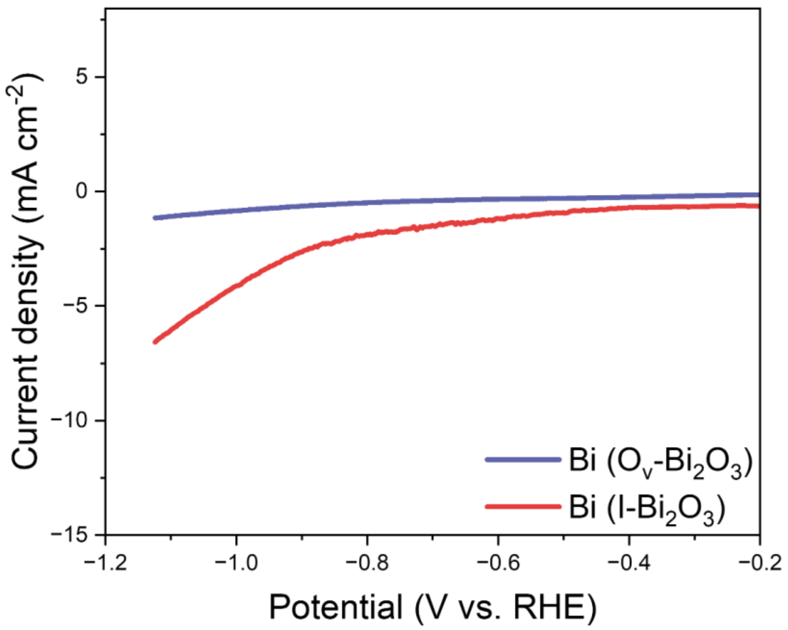

Figure S10. Linear sweep voltammograms for Bi (O_V-Bi_2O_3) and Bi (I-Bi_2O_3) measured at 0.05 mV s^{-1} in 0.2 M N_2-saturated $KHCO_3$.

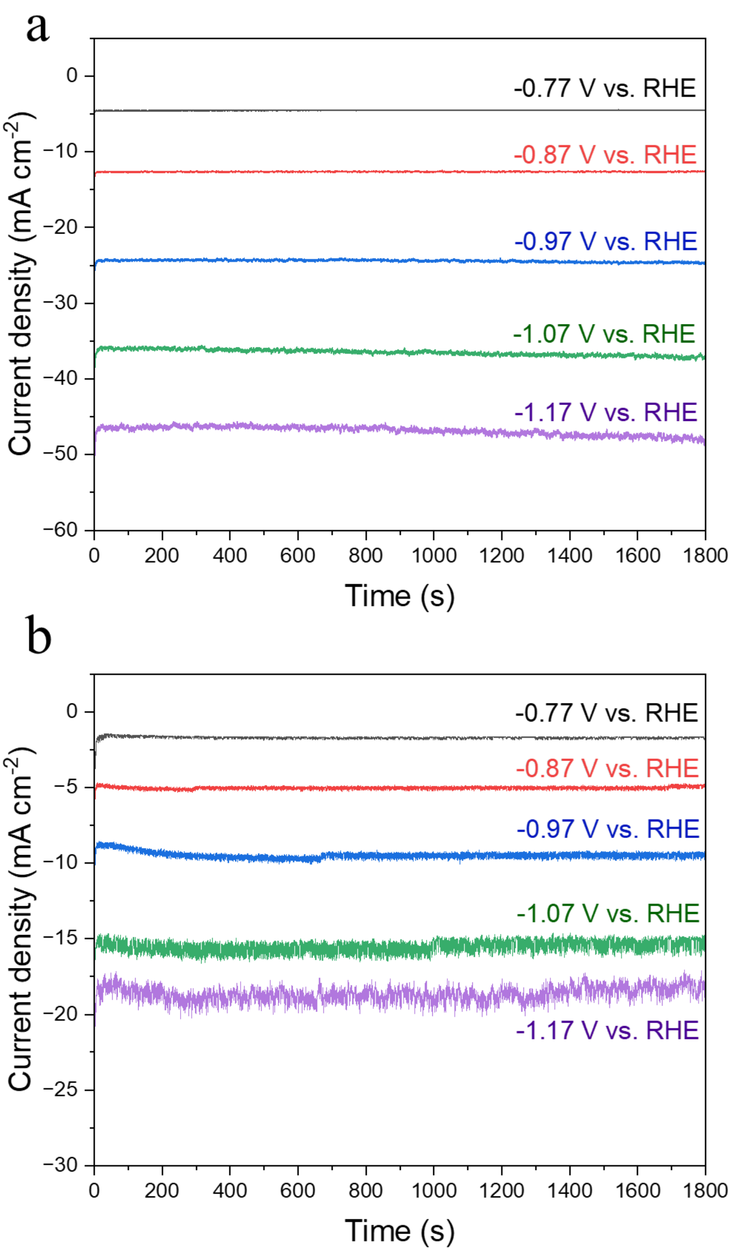

Figure S11. Chronoamperograms of (a) Bi (O_v-Bi_2O_3) and (b) Bi (I-Bi_2O_3) in CO_2-saturated 0.2 M $KHCO_3$ electrolyte at different potentials.

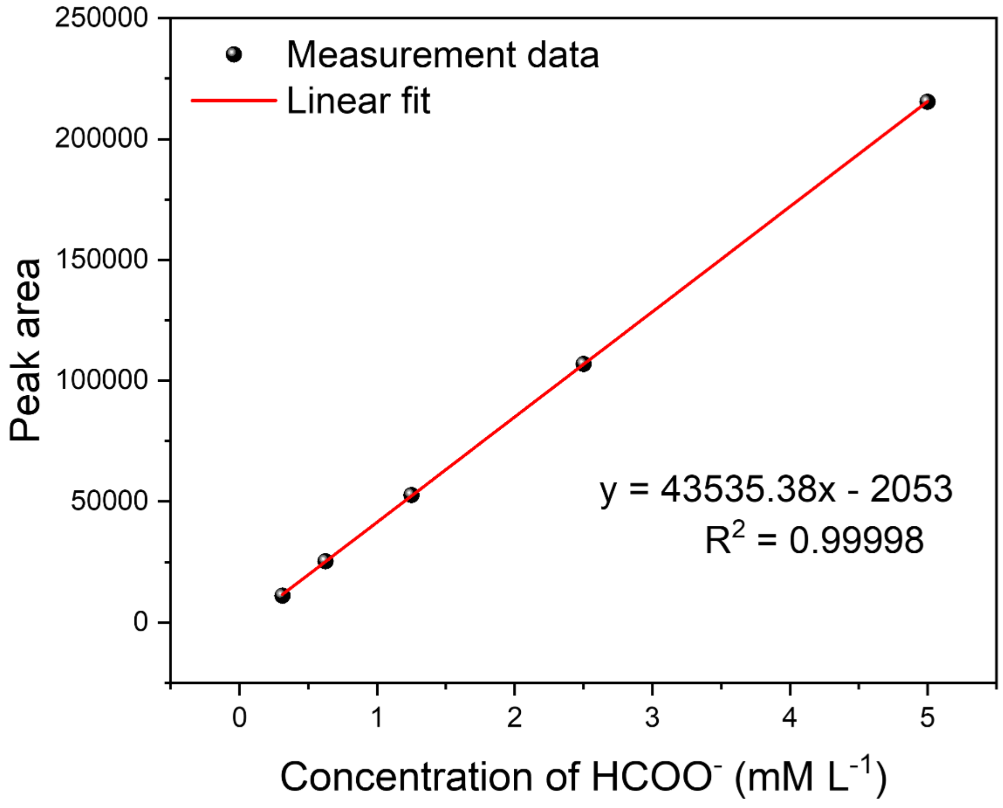

Figure S12. Linear relationship between the known concentrations of $HCOO^-$ and the relative peak area measured by high-performance liquid chromatography.

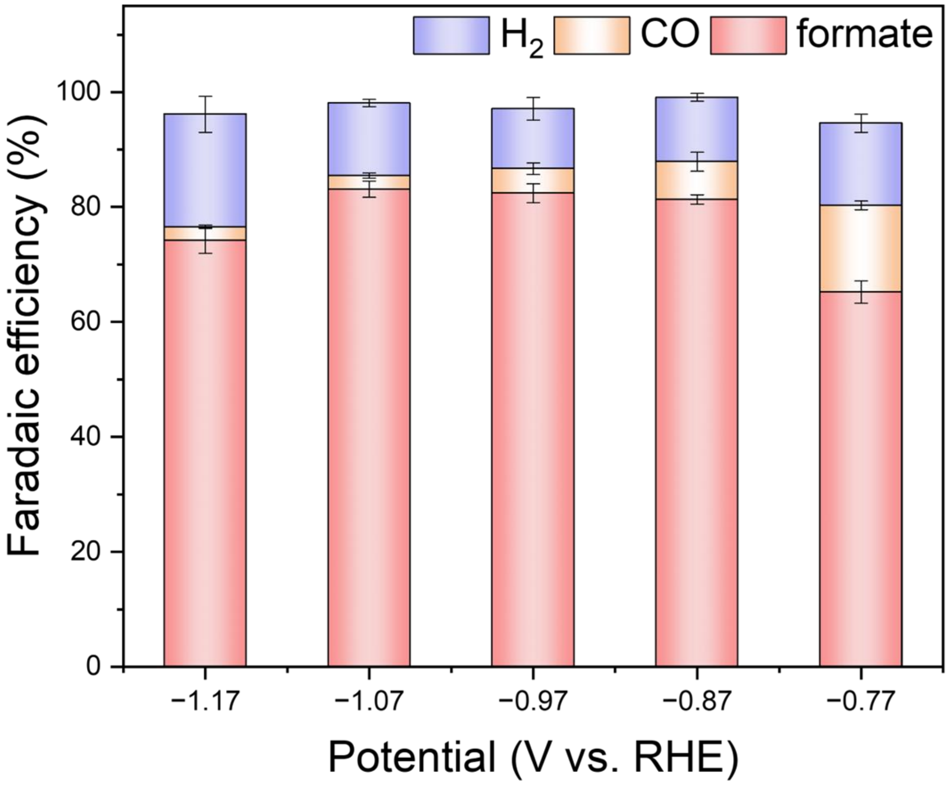

Figure S13. Faradaic efficiencies of $ERCO_2$ products of Bi (I-Bi_2O_3).

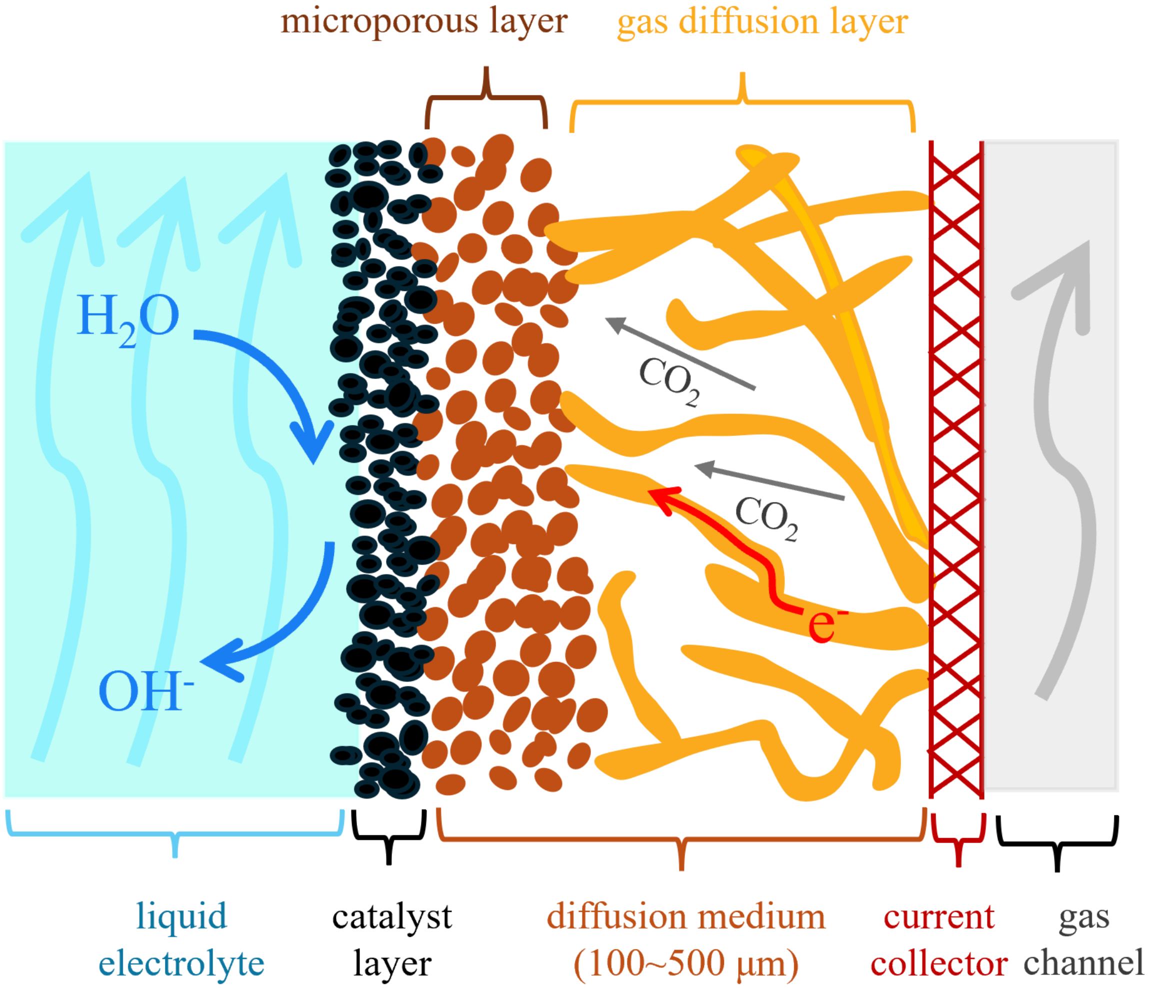

Figure S14. Diagram of the gas diffusion electrode (GDE) configuration for ERCO$_2$.

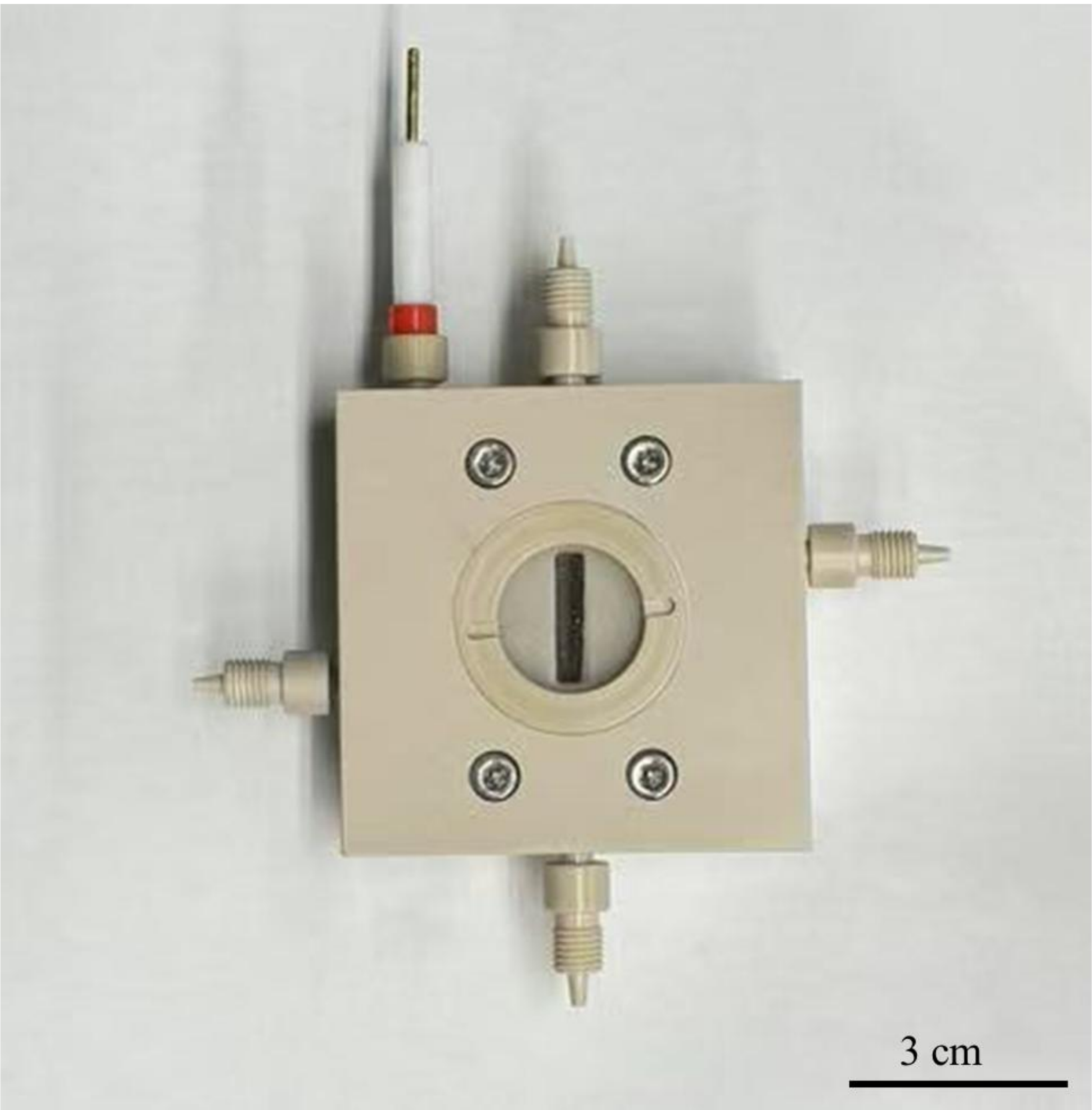

Figure S15. Photograph of flow cell for $ERCO_2$.

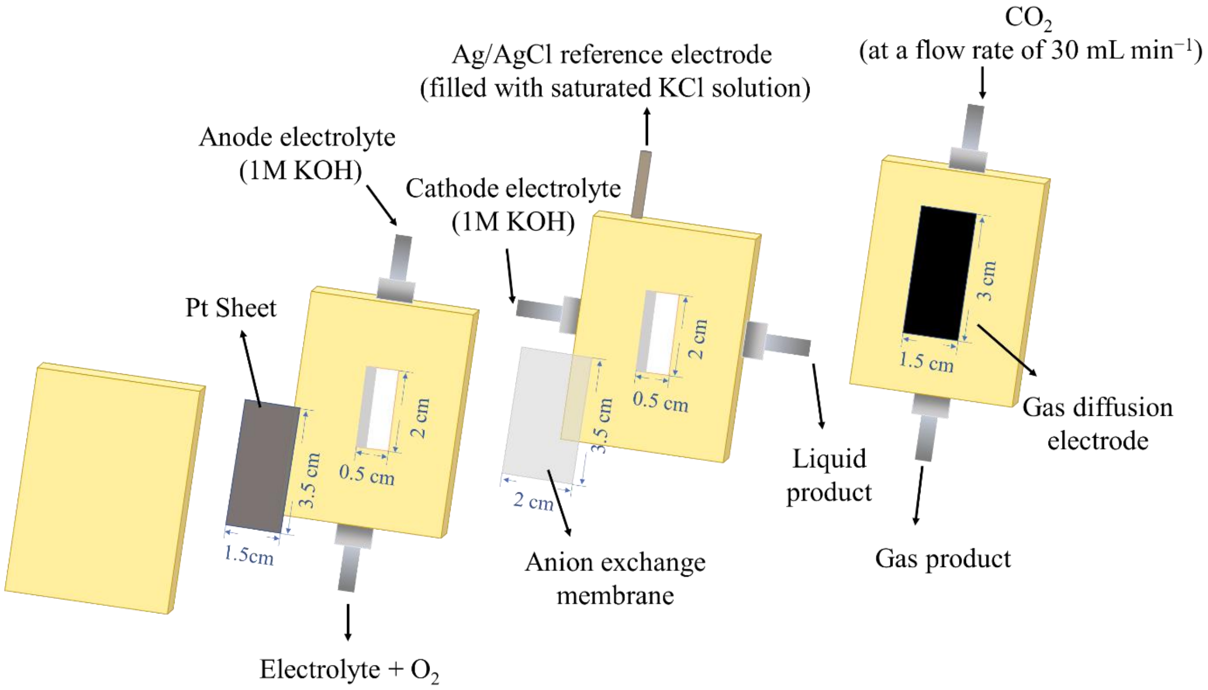

Figure S16. Installation diagram of flow cell for ERCO$_2$.

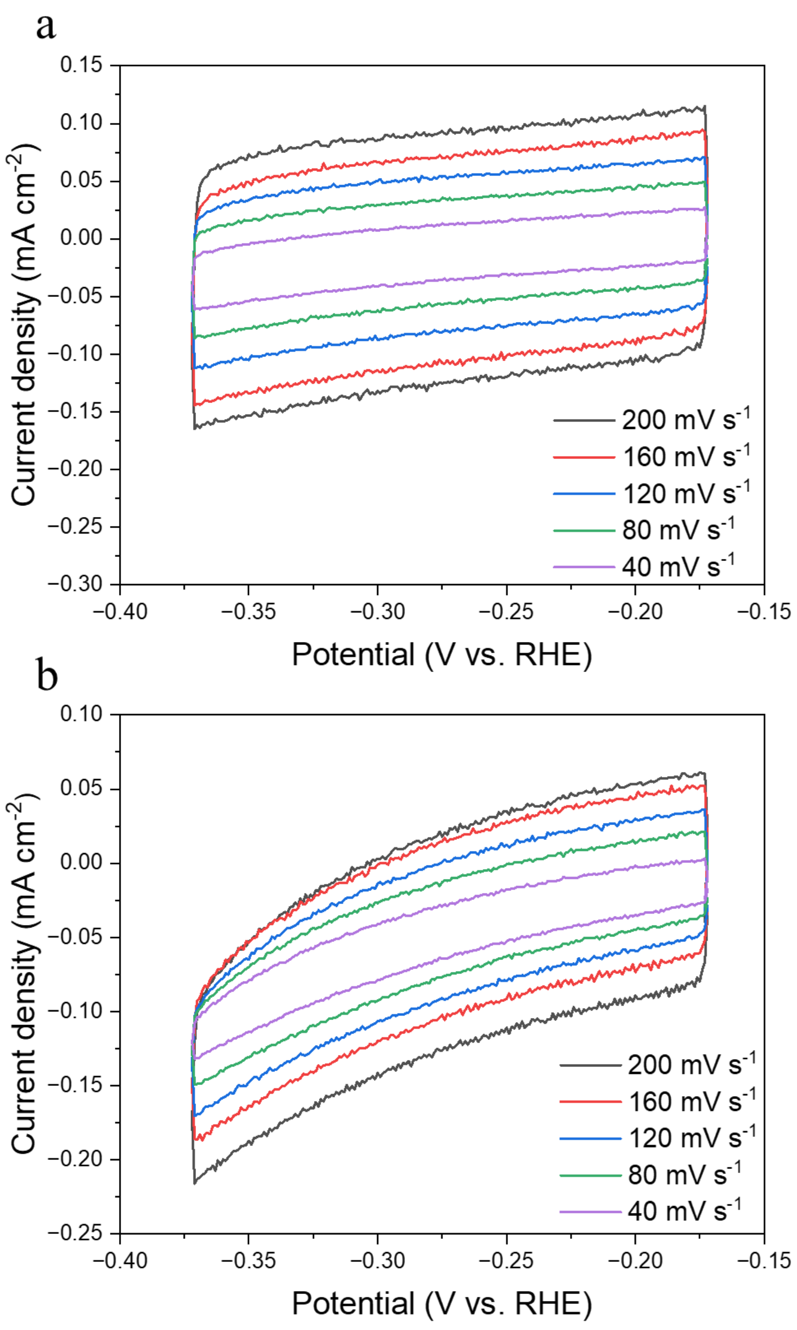

Figure S17. CVs of the catalysts at different sweep speeds: (a) Bi (O_v-Bi_2O_3) and (b) Bi (I-Bi_2O_3).

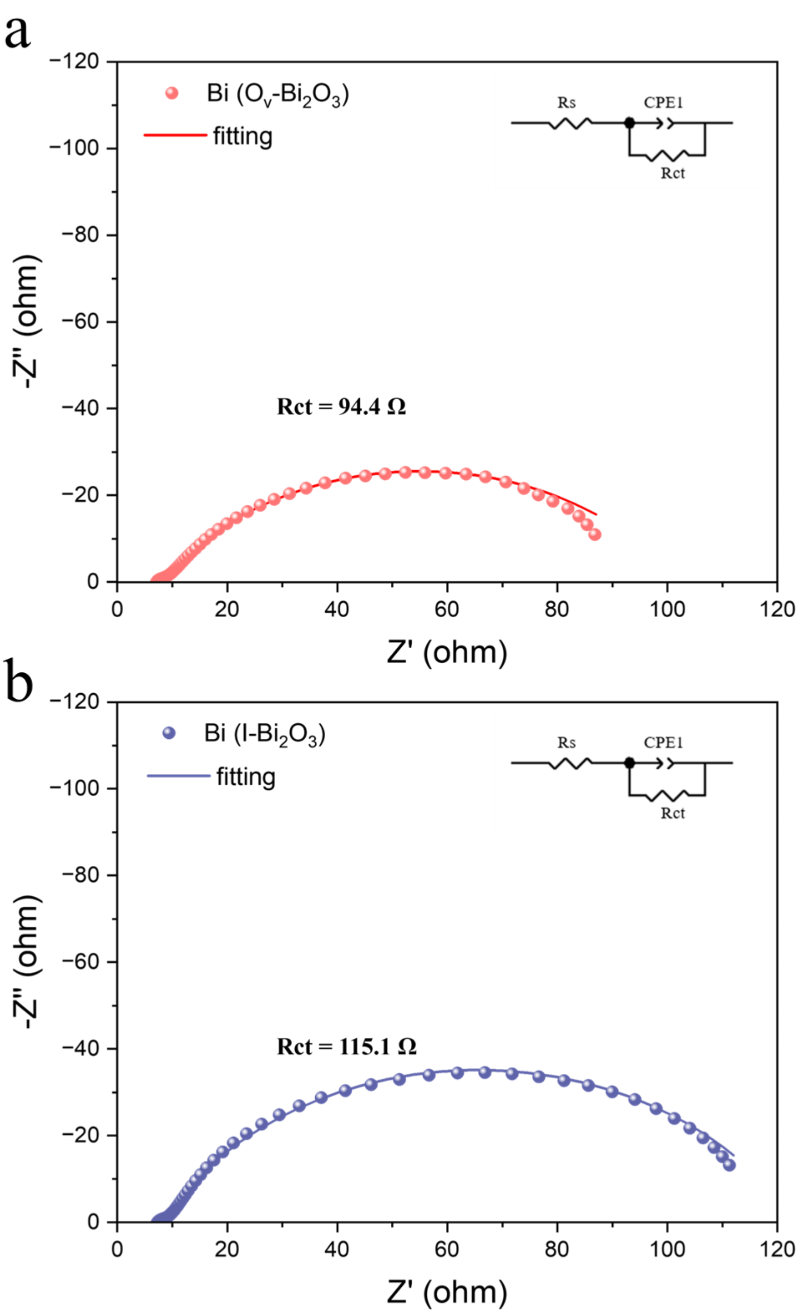

Figure S18. Nyquist plots of (a) Bi (O_v-Bi_2O_3) and (b) Bi (I-Bi_2O_3) (solid symbols are experimental results and solid lines are fitted curves).

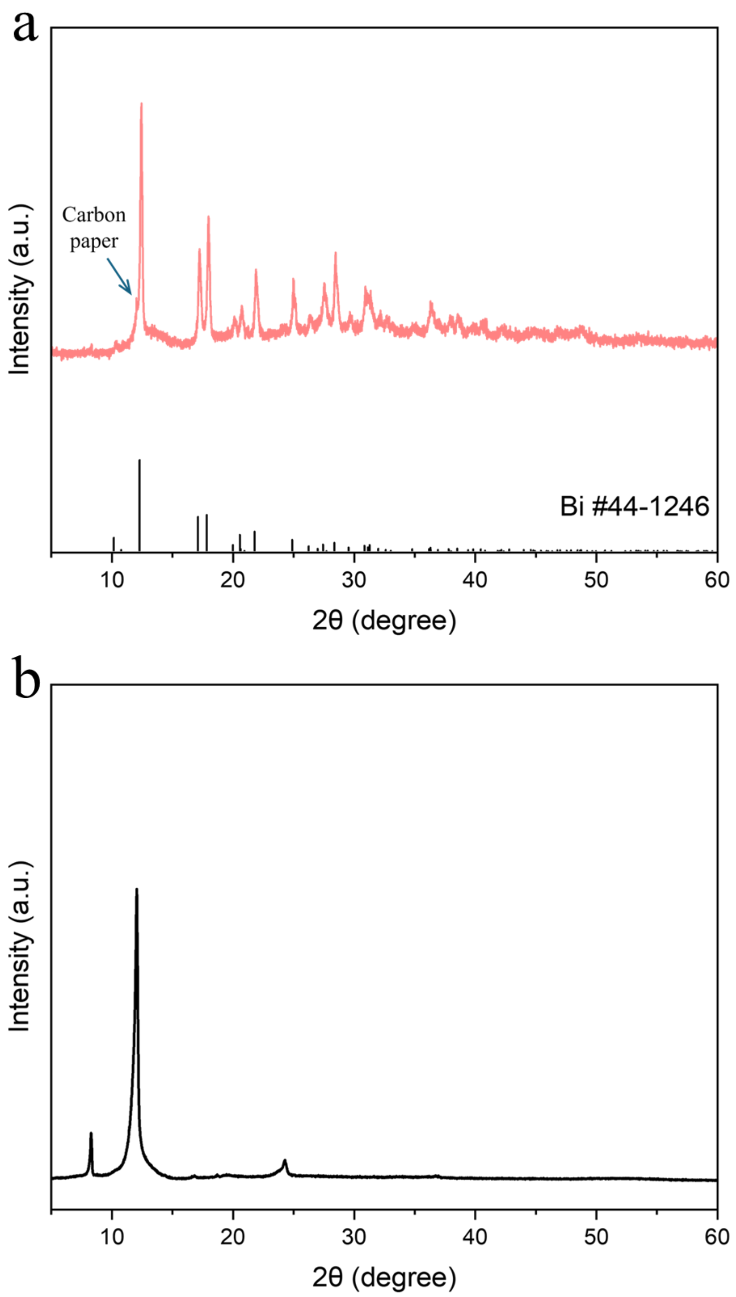

Figure S19. XRD patterns of (a) Bi (O_V-Bi_2O_3) after stability test for 30 h and (b) pristine carbon paper measured with Mo *K*α radiation.

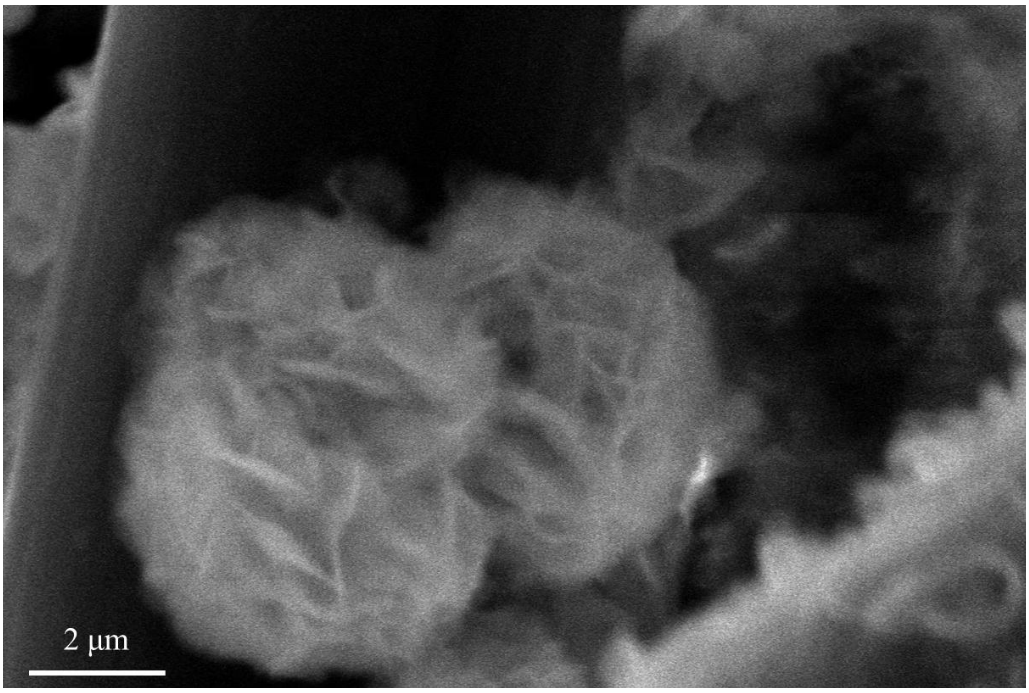

Figure S20. SEM image of Bi (O_v-Bi_2O_3) after stability test for 30 h at −1.07 V vs. RHE in H-type cell with 0.2 M $KHCO_3$ electrolyte.

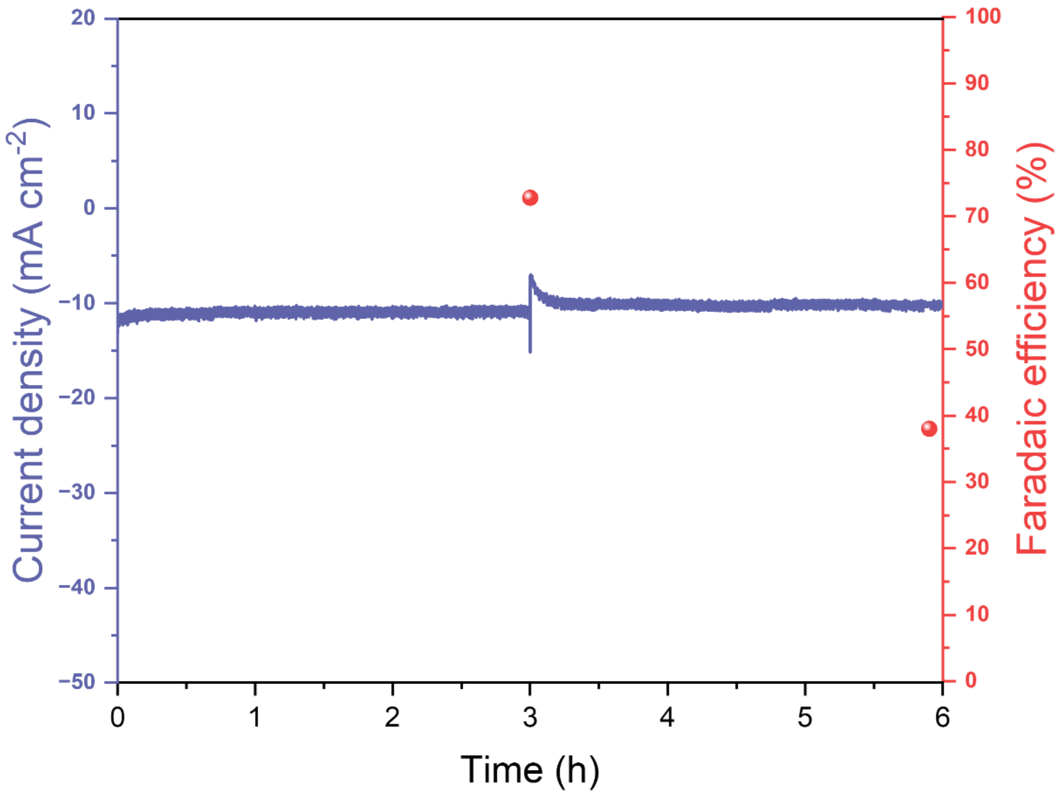

Figure S21. $FE_{formate}$ and current density of Bi (I-Bi_2O_3) during long-term CO_2 electroreduction at –1.07 V vs. RHE for 6 h in H-type cell with 0.2 M $KHCO_3$ electrolyte.

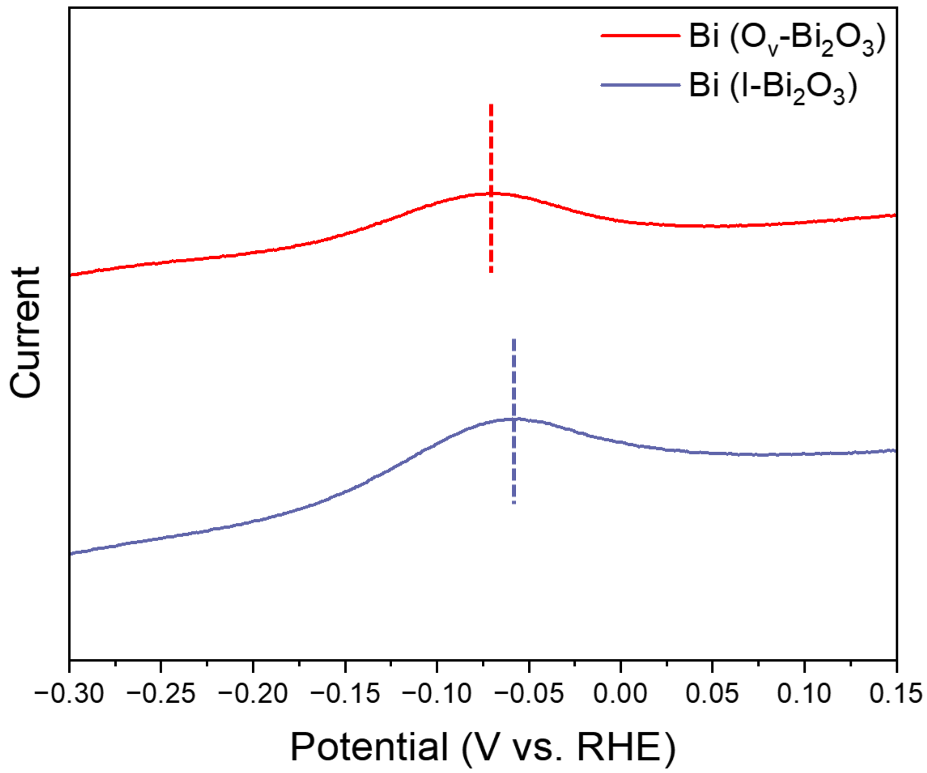

Figure S22. Oxidative LSVs measured in 0.1 M KOH on Bi (O_V-Bi_2O_3) and (b) Bi (I-Bi_2O_3) at scan rate of 0.05 V s^{-1}.

Table S1. Comparison of various Bi-based electrocatalysts for $ERCO_2$ to formate conducted in H-type cell.

Catalysts	Electrolyte	Potential (V vs. RHE)	i (mA cm^{-2})	$FE_{formate}$ (%)	Durability (h)	Ref.
Bi (O_v-Bi_2O_3)	0.2 M $KHCO_3$	–1.07	–36.5	~100	30	This work
Bi-NFs	0.1 M $KHCO_3$	–0.9	–11.4	92.3	16	[1]
Bi NPs/C	0.5 M $KHCO_3$	–0.97	–5.1	95	32	[2]
Bi Nanosheets	0.1 M $KHCO_3$	–1.1	–16.5	86	10	[3]
Bi/rGo	0.1 M $KHCO_3$	–0.8	–2	98	12	[4]
Cu_2BiS_x	0.5 M $KHCO_3$	–0.9	–6.2	98	27.3	[5]
SnO_2/Bi_2O_3	0.1 M $KHCO_3$	–1.0	–3.5	82	12	[6]
$In_{16}Bi_{84}$ NS	0.5 M $KHCO_3$	–0.94	–14.1	~100	10	[7]
In-doped $Bi_2O_2CO_3$ nanosheets	0.5 M $KHCO_3$	–0.9	–9.5	98.3	22	[8]
Bi_2S_3-Bi_2O_3 NSs	0.1 M $KHCO_3$	–1.1	–17.1	93.8	18	[9]
BOC-NS	0.5 M $KHCO_3$	–0.9	–28	98	12	[10]
Bi_2O_3/Bi NPS	0.1 M $KHCO_3$	–1.14	–21.5	88	15	[11]
Porous Bi-Sn	0.1 M $KHCO_3$	–1.00	–9.3	94	10	[12]
np-Sb_2Bi_6 alloy	0.5 M $KHCO_3$	–0.8	–22.26	92	12	[13]

Table S2. Comparison of reported Bi-based catalysts for formate production by $ERCO_2$ in 1 M KOH in flow cell.

Catalysts	Potential (V vs. RHE)	i (mA cm^{-2})	$FE_{formate}$ (%)	formation rate (mmol h^{-1} cm^{-2})	Ref.
Bi (O_v-Bi_2O_3)	−1.08	−340	96	4.7	This work
Pits-Bi	−1.4	−325	>95	6.1	[14]
Bi-ene	−0.75	−200	99.2	3.7	[15]
Bi-NSs	−0.67	−405	89	7.5	[16]
Bi_2S_3-Bi_2O_3 NSs	−1.0	−145	95.3	2.7	[9]
Bi rhombic dodecahedrons	−0.78	−279	93	5.2	[17]
Bi LNSs	−1.1	−590	92.2	11	[18]
Bi_2O_3-CeO_x	−1.10	−205	97.7	3.8	[19]
np-Sb_2Bi_6 alloy	−0.91	−382	95.6	7.1	[13]
Bi_2O_3@C/HB	−1.1	−285	95	5.3	[18]
Sn-doped Bi/BiO_x	−1.0	−256	85	4.7	[20]

References

[1] S. Yang, M. Jiang, W. Zhang, Y. Hu, J. Liang, Y. Wang, Z. Tie, Z. Jin, *Adv. Funct. Mater.* **2023**, 33, 2301984.

[2] C. Cao, D.-D. Ma, J. Jia, Q. Xu, X.-T. Wu, Q.-L. Zhu, *Adv. Mater.* **2021**, 33, 2008631.

[3] W. Zhang, Y. Hu, L. Ma, G. Zhu, P. Zhao, X. Xue, R. Chen, S. Yang, J. Ma, J. Liu, Z. Jin, *Nano Energy* **2018**, 53, 808.

[4] Y.-X. Duan, K.-H. Liu, Q. Zhang, J.-M. Yan, Q. Jiang, *Small Methods* **2020**, 4, 1900846.

[5] X. Yang, Q. Wang, F. Chen, H. Zang, C. Liu, N. Yu, B. Geng, *Nano Res.* **2023**,16, 7974.

[6] J. Tian, R. Wang, M. Shen, X. Ma, H. Yao, Z. Hua, L. Zhang, *ChemSusChem* **2021**, 14, 2247.

[7] D. Tan, W. Lee, Y. E. Kim, Y. N. Ko, M. H. Youn, Y. E. Jeon, J. Hong, J. E. Park, J. Seo, S. K. Jeong, Y. Choi, H. Choi, H. Y. Kim, K. T. Park, *ACS Appl. Mater. Interfaces* **2022**, 14, 28890.

[8] M. Wu, Y. Xiong, B. Hu, Z. Zhang, B. Wei, L. Li, J. Hao, W. Shi, *J. Colloid Interface Sci.* **2022**, 624, 261.

[9] P.-F. Sui, C. Xu, M.-N. Zhu, S. Liu, Q. Liu, J.-L. Luo, *Small* **2022**, 18, 2105682.

[10] T. Fan, W. Ma, M. Xie, H. Liu, J. Zhang, S. Yang, P. Huang, Y. Dong, Z. Chen, X. Yi, *Cell Rep. Phys. Sci.* **2021**, 2, 100353.

[11] T. Tran-Phu, R. Daiyan, Z. Fusco, Z. Ma, R. Amal, A. Tricoli, *Adv. Funct. Mater.* **2020**, 30, 1906478.

[12] Z. Wu, H. Wu, W. Cai, Z. Wen, B. Jia, L. Wang, W. Jin, T. Ma, *Angew. Chem. Int. Ed.* **2021**, 60, 12554.

[13] F. Pan, X. Yang, T. O'Carroll, H. Li, K.-J. Chen, G. Wu, *Adv. Energy Mater.* **2022**, 12, 2200586.

[14] Y. Yuan, Q. Wang, Y. Qiao, X. Chen, Z. Yang, W. Lai, T. Chen, G. Zhang, H. Duan, M. Liu, H. Huang, *Adv. Energy Mater.* **2022**, 12, 2200970.

[15] S. Verma, Y. Hamasaki, C. Kim, W. X. Huang, S. Lu, H. R. M. Jhong, A. A. Gewirth, T. Fujigaya, N. Nakashima, P. J. A. Kenis, *Acs Energy Lett* **2018**, 3, 193.

[16] J. Yang, X. Wang, Y. Qu, X. Wang, H. Huo, Q. Fan, J. Wang, L.-M. Yang, Y. Wu, *Advanced Energy Materials* **2020**, 10, 2001709.

[17] H. Xie, T. Zhang, R. Xie, Z. Hou, X. Ji, Y. Pang, S. Chen, M. M. Titirici, H. Weng, G. Chai, *Adv. Mater.* **2021**, 33, e2008373.

[18] D. Wang, C. Liu, Y. Zhang, Y. Wang, Z. Wang, D. Ding, Y. Cui, X. Zhu, C. Pan, Y. Lou, F. Li, Y. Zhu, Y. Zhang, *Small* **2021**, 17, 2100602.

[19] R. Yu, C. Qiu, Z. Lin, H. Liu, J. Gao, S. Li, Y. Yao, J. Yu, S. Yang, *ACS Mater. Lett.* **2022**, 4, 1749.

[20] Y. Zhao, X. Liu, Z. Liu, X. Lin, J. Lan, Y. Zhang, Y.-R. Lu, M. Peng, T.-S. Chan, Y. Tan, *Nano Lett.* **2021**, 21, 6907.
